# A spatial sequencing atlas of age-induced changes in the lung during influenza infection

**DOI:** 10.1038/s41467-023-42021-y

**Published:** 2023-10-18

**Authors:** Moujtaba Y. Kasmani, Paytsar Topchyan, Ashley K. Brown, Ryan J. Brown, Xiaopeng Wu, Yao Chen, Achia Khatun, Donia Alson, Yue Wu, Robert Burns, Chien-Wei Lin, Matthew R. Kudek, Jie Sun, Weiguo Cui

**Affiliations:** 1 Department of Microbiology and Immunology, Medical College of Wisconsin, Milwaukee, WI 53226 USA; 2 Blood Research Institute, Versiti Wisconsin, Milwaukee, WI 53226 USA; 3 Department of Pediatrics, Medical College of Wisconsin, Milwaukee, WI 53226 USA; 4 Carter Immunology Center, University of Virginia, Charlottesville, VA 22908 USA; 5 Division of Infectious Diseases and International Health, University of Virginia, Charlottesville, VA 22908 USA; 6 Department of Biostatistics, Medical College of Wisconsin, Milwaukee, WI 53226 USA; 7 Department of Pathology, Northwestern University, Chicago, IL 60611 USA

**Keywords:** Influenza virus, RNA sequencing, Adaptive immunity, Ageing

## Abstract

Influenza virus infection causes increased morbidity and mortality in the elderly. Aging impairs the immune response to influenza, both intrinsically and because of altered interactions with endothelial and pulmonary epithelial cells. To characterize these changes, we performed single-cell RNA sequencing (scRNA-seq), spatial transcriptomics, and bulk RNA sequencing (bulk RNA-seq) on lung tissue from young and aged female mice at days 0, 3, and 9 post-influenza infection. Our analyses identified dozens of key genes differentially expressed in kinetic, age-dependent, and cell type-specific manners. Aged immune cells exhibited altered inflammatory, memory, and chemotactic profiles. Aged endothelial cells demonstrated characteristics of reduced vascular wound healing and a prothrombotic state. Spatial transcriptomics identified novel profibrotic and antifibrotic markers expressed by epithelial and non-epithelial cells, highlighting the complex networks that promote fibrosis in aged lungs. Bulk RNA-seq generated a timeline of global transcriptional activity, showing increased expression of genes involved in inflammation and coagulation in aged lungs. Our work provides an atlas of high-throughput sequencing methodologies that can be used to investigate age-related changes in the response to influenza virus, identify novel cell-cell interactions for further study, and ultimately uncover potential therapeutic targets to improve health outcomes in the elderly following influenza infection.

## Abstract (web summary)

Ageing is known to impair the immune response against infectious pathogens. Here, Kasmani et al. present a spatial and transcriptomic atlas of immune changes in the lungs of young and aged mice in response to influenza virus infection.

## Introduction

Influenza A virus (IAV) is a negative-strand RNA virus capable of genetic reassortment that seasonally causes respiratory tract infections^1^. Infections with IAV are generally nonlethal; however, certain patient populations are at high risk of mortality, including young children, the immunocompromised, and the elderly^2^. Increased age is strongly correlated with both increased morbidity and mortality following influenza virus infection^3^, as aging impairs both the human immune response to natural influenza infection and the anamnestic response to influenza vaccination^4–7^. Murine models of aging have been shown to have qualitatively and quantitively impaired anti-influenza immune responses: aged lungs exhibit altered myeloid cell recruitment and function;^8,9^ lower titers of neutralizing antibodies;^10^ and delayed and diminished recruitment of CD4 and CD8 T cells to the lung during infection, which results in delayed viral clearance^11–13^. Aged mice also have impaired memory T cell responses, with tissue-resident memory (TRM) CD8 T cells in aged mice exhibiting impaired antiviral recall functionality and instead promoting lung fibrosis following infection resolution^14^.

The lungs and airways are comprised of several different types of epithelial cells with specialized functions, including type 1 (AT1) and type 2 (AT2) pneumocytes; goblet, ciliated, and club cells; and a variety of fibroblasts^15^. In addition, the lungs are a highly vascularized site, with dense capillary networks necessary to support gas exchange^16^. Two recent studies have used scRNA-seq to identify a novel population of endothelial cells that promote vascular regeneration in the murine lung^17,18^, and another has identified vascular heterogeneity in the human lung^19^, emphasizing the vast cellular heterogeneity of both epithelial and endothelial cells in the lungs.

Aging alters all organ systems, not just the immune system; as such, aging may also impact the host response to IAV through direct changes in lung tissue. As a result, recent studies have begun to investigate interactions between systems in the respiratory tract, often by using scRNA-seq^20^. Applications of scRNA-seq to young and aged mice have identified age-induced changes that occur at homeostasis^21^. Our lab and others have shown that aged immune cells drive a senescent phenotype in non-immune tissues^22^, that aged pulmonary epithelial cells recruit greater numbers of immune cells to the lung via excessive chemokine production post-IAV infection^8^, and that a subset of type 2 innate lymphoid cells (ILC2s) promotes wound healing and tissue repair in the lung following IAV infection^23^. These findings, among several others^20^, all highlight the importance of crosstalk among immune, epithelial, and endothelial cells during infection. However, further work is needed to characterize these cell-cell interactions and how they may be disrupted in aged hosts following influenza infection.

One particular avenue in need of further investigation is that of cellular localization. The ability of cells, particular immune cells, to mediate their functions and interact with other cell types is contingent upon their ability to appropriately localize to the region of interest. Fluorescence microscopy is a common technique for identifying cell-cell colocalization. However, new spatial sequencing techniques, such as the 10x Genomics Visium platform, offer the ability to identify cells and their gene expression profiles in situ at a resolution not previously possible, particularly when paired with scRNA-seq^24–26^. Spatial transcriptomics therefore offers a high throughput method to characterize changes in cellular localization and cell-cell interactions, thus allowing for the identification of novel pathways that may ultimately be targeted for therapeutic benefit.

The aforementioned observations collectively suggest that immune-epithelial-endothelial crosstalk in the lung is critical during influenza virus infection, and that these interactions may be altered by aging. Improved understanding of these interactions will be critical for understanding the pathology of influenza virus infection and may have implications for the infection course of other respiratory viruses, such as SARS-CoV-2 and emerging respiratory viral pathogens. Therefore, we sought to utilize a combination of scRNA-seq, spatial sequencing, and bulk RNA-seq to generate a multifaceted, high-resolution atlas of young and aged lung tissue following influenza infection and identify key changes induced by aging in this clinically relevant setting.

## Results

### Single-cell RNA sequencing reveals cellular heterogeneity among young and aged lungs post-influenza infection

**Fig. 1:**
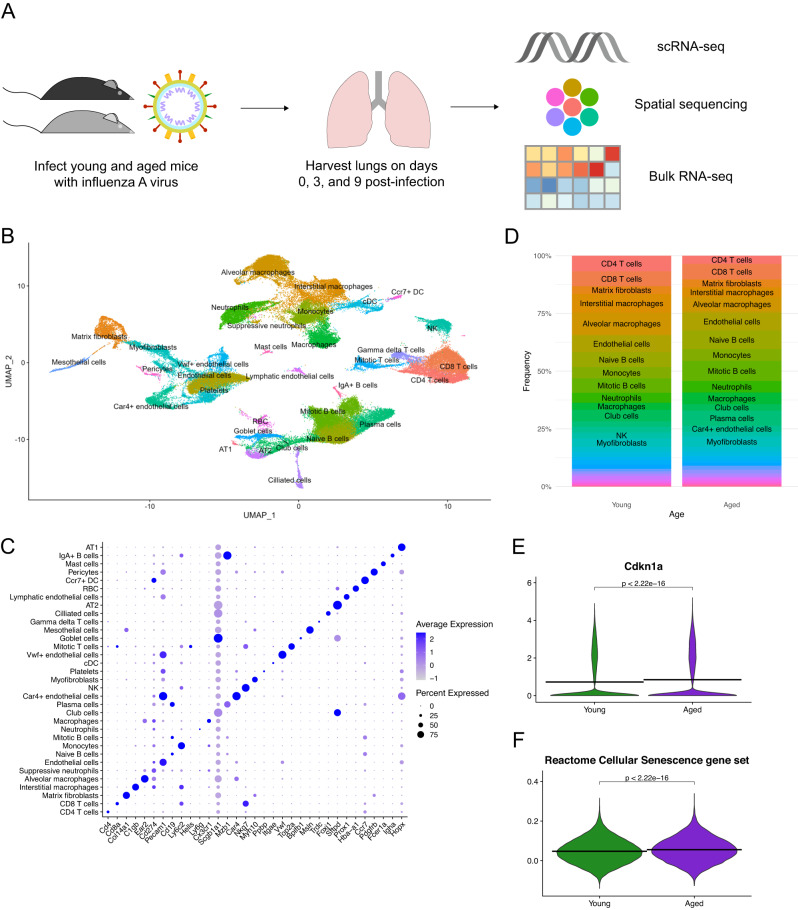
Single-cell RNA sequencing reveals cellular heterogeneity among young and aged lungs post-influenza infection. **A** Schematic of experimental design. **B** UMAP plot of scRNA-seq data, with each of 33 clusters labeled. Each dot represents one of 82,079 cells. **C** Dot plot showing key markers used to identify cluster identities. Color denotes expression level, dot size denotes percentage of cells in each cluster expressing a given gene. **D** Bar plot showing relative frequency of cell types by age group. **E** Violin plot showing expression of *Cdkn1a* (encodes p14) between young and aged samples. **F** Violin plot showing module scores of the Reactome Cellular Senescence gene set (GSEA systematic name M27188). Horizontal lines denote mean values. Statistical testing was performed using the two-sided Wilcoxon test without multiple comparison correction. Data in (**B**–**F**) are pooled from all scRNA-seq data (day 0, day 3, and day 9). See also Supplemental Table 1.

In order to investigate age-induced alterations in the host response to influenza A virus (IAV) infection, we infected groups of three young (16–18-week-old) and three aged (80–82-week-old) female C57Bl/6 mice intranasally with 50 PFU of the PR8 H1N1 variant of IAV. Lungs were harvested on day 3 or day 9 post-infection and processed for single-cell RNA sequencing (scRNA-seq), Visium spatial sequencing, or bulk RNA sequencing (bulk RNA-seq) (Fig. 1A, Supplemental Fig. 1A, B, and Methods). We also used the R package Seurat to integrate^27,28^ our 12 scRNA-seq samples with published scRNA-seq data from naïve, uninfected young and aged mice^29^. After integrating all 12 samples (day 0, day 3, and day 9 post-infection) and performing quality control to exclude doublets and dead cells, we were left with 82,079 cells that formed 33 clusters using uniform manifold approximation and projection (UMAP) visualization (Fig. 1B). We used this combined scRNA-seq dataset for all further analyses unless otherwise specified. As we performed scRNA-seq on all cells in the lung, we found clusters consisting of immune cells, epithelial and stromal cells, and endothelial cells. To identify each cluster, we constructed a dot plot of key marker genes for these various cell types based on published scRNA-seq data^17–19,29,30^ (Fig. 1C). We then used a bar plot to quantify relative frequencies of these clusters between young and aged mice pooled from all timepoints (Fig. 1D and Supplemental Table 1).

Aging is known to be associated with senescence, a cellular state marked by lack of proliferation and resistance to apoptosis^31^. To examine whether our data was able to capture these changes, we examined global expression of *Cdkn1a*, which encodes the senescence marker p21^32^. As might be expected, we found that cells pooled all timepoints of scRNA-seq data from the lungs of aged mice expressed significantly higher levels of *Cdkn1a* than did those of young mice pooled from all timepoints (Fig. 1E). To verify this, we wanted to use several markers rather than simply one to determine the degree of cellular senescence. As such, we calculated module scores of the Reactome Cellular Senescence gene set (GSEA systematic name M27188). Leveraging over 100 senescence markers, we once again found that aged mice expressed significantly higher levels of genes in this gene set than young mice (Fig. 1F). Collectively, these preliminary analyses demonstrated the power afforded by scRNA-seq in conjunction with kinetically staggered samples to identify age-induced and temporal changes in various cell types in the lung in response to influenza infection.

### Spatial sequencing allows for topological analysis of cellular and systemic processes altered by aging

**Fig. 2:**
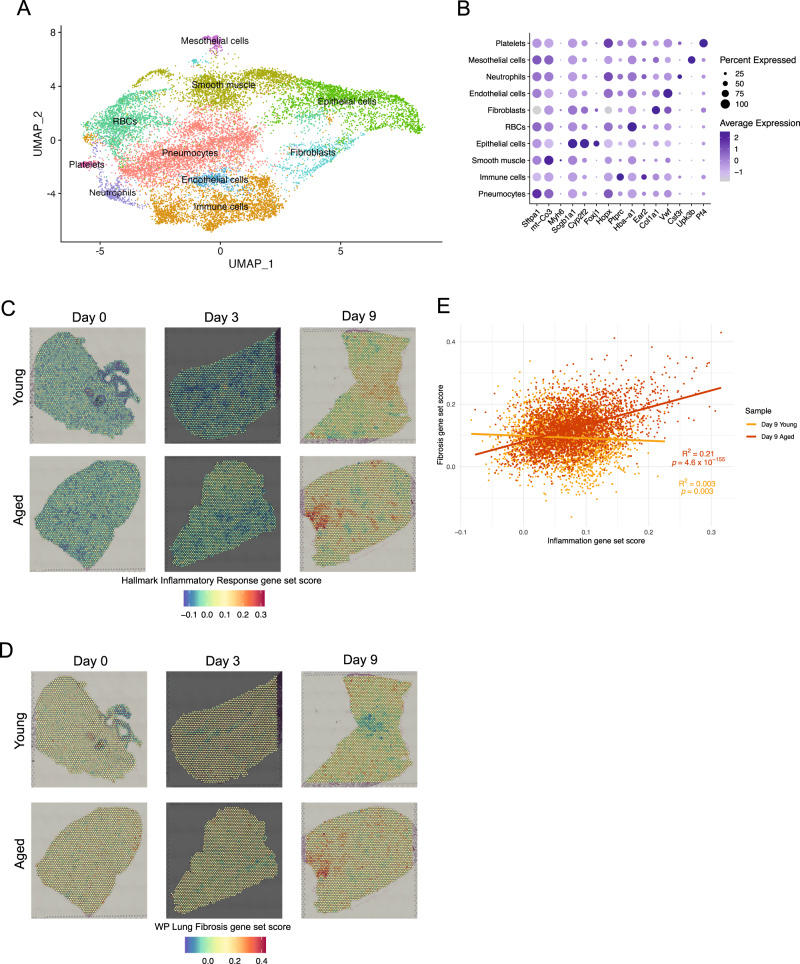
Spatial sequencing allows for topological analysis of cellular and systemic processes altered by aging. **A** UMAP plot of Visium data, with each of 10 clusters labeled. Each dot represents one of 15,026 capture spots. **B** Dot plot showing key markers used to identify cluster identities. Color denotes expression level, dot size denotes percentage of capture spots in each cluster expressing a given gene. **C** Spatial feature plots showing scores across lung sections of the Hallmark Inflammatory Response gene set (GSEA systematic name M5932). **D** As in (**C**), but using the WP Lung Fibrosis gene set (GSEA systematic name M39477). **E** Linear regression between inflammation and fibrosis gene sets from young and aged lungs day 9 post-infection shown in (**C**) and (**D**). Each point denotes one capture spot, color denotes sample identity. Statistical testing was performed using a two-sided t-test without multiple comparison correction.

It is understood that localization of cells plays an important role in regulating cell-cell interactions in both physiological and pathological processes. Although traditional spatial visualization methods such as immunohistochemistry or fluorescence microscopy allow for identification of proteins, they are limited in the number of markers that can be concomitantly detected in a given tissue section and these markers must be chosen a priori. Therefore, we opted to use the 10x Genomics Visium spatial RNA sequencing platform to generate high throughput transcriptomic captures of lungs from young and aged mice at day 0 (naïve), day 3, and day 9 post-infection with PR8 IAV (Fig. 1A). Visium slides allow for simultaneous viewing of hematoxylin and eosin (H&E)-stained histology (Supplemental Fig. 2A) as well as 55 micron diameter capture spots of pooled captured mRNA (Supplemental Fig. 2B–D). These capture spots are individually barcoded, allowing us to identify putative cell types based on mRNA expression within each spot across the tissue section. Similar to scRNA-seq, in which dimensionality reduction is used to cluster individual cells, we used UMAP visualization to visualize and cluster 15,026 individual capture spots from all six lung samples (Fig. 2A). Using key markers, we were able to identify the major cell type in each cluster (Fig. 2B): pneumocytes, immune cells, smooth muscle, epithelial cells, red blood cells (RBCs), fibroblasts, endothelial cells, neutrophils, mesothelial cells, and platelets. Notably, the 55 micron resolution afforded by Visium spatial sequencing does not allow for single-cell resolution. As a result, each of our spatial sequencing clusters tended to express low levels of markers of other clusters in addition to their own respective key marker; for example the immune cell cluster most strongly expressed *Ptprc* (encodes CD45), but many of the capture spots in this cluster also expressed *Sftpa1* (encodes surfactant protein A1, which is produced by type 2 pneumocytes (AT2 cells)) (Fig. 2B). This stands in contrast to our scRNA-seq data, in which most marker genes were uniquely expressed by their respective clusters (Fig. 1C). To improve on the multicellular resolution available with Visium, we used the SPOTlight package in R to predict the cellular composition of each Visium spot using our scRNA-seq data as a reference. ^33^. SPOTlight generated a pie chart from each spot of our Visium capture spots, allowing us to later correlate putative locations of specific cell types with other parameters captured by our spatial sequencing data (Supplemental Fig. 2B–D).

To demonstrate the enormous analytical power afforded by spatial sequencing, we used module scores to identify upregulation of key biological processes in a cell type-agnostic spatial manner. We began by visualizing module scores of the Hallmark Inflammatory Response gene set (GSEA systematic name M5932) as a way to identify where inflammation occurred in each lung tissue section (Fig. 2C). As expected, inflammation was low in naïve lungs but increased at day 3 and dramatically increased at day 9 post-infection. We also quantified the inflammation module score in each tissue section and found a significant increase in inflammation in aged lungs compared to young lungs at all three timepoints (Supplemental Fig. 2E). This agrees with prior observations that aged mice tend to have increased inflammatory responses during influenza infection^10^. This increased level of inflammation is also known to occur at baseline before pathogen challenge, a condition referred to as inflammaging^34^. Given that aged mice undergo greater tissue fibrosis following IAV infection compared to young mice^14^, we also checked scores of the WP Lung Fibrosis gene set (GSEA systematic name M39477) in our spatial sequencing data (Fig. 2D). As with inflammation, fibrosis scores were highest on day 9 post-infection and were consistently and significantly elevated in aged mice (Supplemental Fig. 2F). It was also apparent that the center-left section of the aged day 9 lung tissue section scored highly for both inflammation and fibrosis gene sets, a topological correlation that would be impossible to detect by bulk RNA-seq or scRNA-seq. To quantify this, we performed a linear regression analysis between the inflammation and fibrosis gene sets in the lungs of young and aged mice at day 9 post-IAV infection (Fig. 2E); in this analysis, each point represented one capture spot from these two tissue sections. We found a strong and significant positive correlation between inflammation and fibrosis module scores on a per-capture spot basis in aged lung tissue. In contrast, young lung tissue had a very weak negative correlation between inflammation and fibrosis. These data suggest that inflammation promotes fibrosis in the lungs of aged mice during IAV infection, whereas young mice are able to prevent the development of fibrosis in inflamed regions.

### Neutrophils in aged mice exhibit altered tissue localization and chemotactic gene expression

Neutrophils mediate inflammation in a variety of pathophysiological processes and are known to drive age-associated mortality following IAV infection^8^. Given this, we turned to our scRNA-seq data to identify temporal and age-related changes in the neutrophil population during IAV infection (Supplemental Fig. 3A). We noticed that genes encoding neutrophil granule proteins such as *Ltf* (lactotransferrin), *Camp* (cathelicidin antimicrobial peptide), and *Ngp* (neutrophilic granule protein) were all expressed at significantly higher levels pre-infection in young neutrophils compared to aged neutrophils. However, aged neutrophils did upregulate the proinflammatory gene *Il1b* (encodes IL-1β) at baseline. At day 3 post-infection, young and aged neutrophils expressed similar levels of *Il1b*, but young neutrophils expressed higher levels of *Il1a*; the chemokines *Ccl3*, *Cxcl3*, and *Cxcl1*; and *Cd274* (encodes PD-L1). Neutrophils from mice of both age groups also upregulated common genes at this timepoint such as *Bcl2a1b* (encodes the anti-apoptotic protein BCL-2 A1)^35^, *Traf1*, and *Sod2* (encodes superoxide dismutase 2).

As gene expression analysis suggested that pro-inflammatory genes are differentially expressed between young and aged neutrophils at different timepoints post-infection, we quantified these functional changes using module scores. The inflammatory module score was significantly higher in aged neutrophils at baseline but lower or comparable in aged neutrophils following infection (Supplemental Fig. 3B). Collectively, these trends suggested that neutrophils from aged mice may have impaired inflammatory function on a per cell basis. Although this initially seemed to contradict aforementioned data that aged mice undergo increased inflammation during IAV infection (Fig. 2C and Supplemental Fig. 2E)^10^, we reasoned that neutrophils in aged mice could make up for inflammatory quality with cellular quantity, as has been demonstrated previously^8^.

**Fig. 3:**
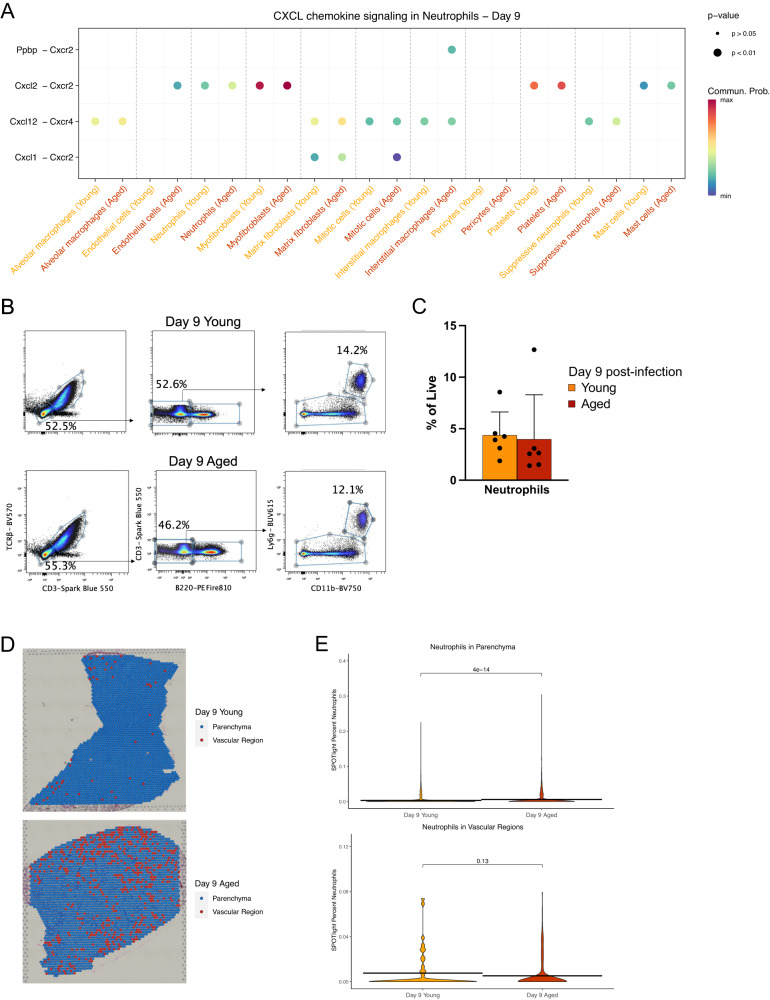
Neutrophils in aged mice exhibit altered chemotactic gene expression and tissue localization. **A** Dot plot showing predicted interactions between CXC chemokine ligands produced by cells labeled on the x-axis and CXC chemokine receptors located on neutrophils at day 9 post-infection. Color denotes communication probability, size denotes *p*-value. **B**, **C** Representative flow plots **B** and frequencies **C** of CD11b^+^ Ly6G^+^ neutrophils on day 9 post-infection in young and aged lungs. **D** Visium plots showing locations of vascular and parenchymal regions of lung based on vascular gene expression. **E** Violin plots comparing neutrophil localization in vascular and parenchymal regions between young and aged mice, based on (**D**). Statistical testing was performed using a two-sided Wilcoxon test without multiple comparison correction (**A**–**E**) or two-sided two-sample *t*-test (**C**). Error bars in (**C**) denote mean ± standard deviation. Flow cytometry data are pooled from two independent experiments, *n* = 3 mice per age group per experiment. Source data are provided as a Source Data file.

As chemokine signaling is known to play a major role in neutrophil recruitment to the inflamed lung during influenza infection, especially via CXCL2-CXCR2 interactions^36^, we turned our attention to the impact of aging on neutrophil chemotaxis. We used the R package CellChat^37^ to predict CXCL chemokine signaling to neutrophils from all cell populations found in our scRNA-seq data. At day 3 post-infection, young neutrophils were consistently predicted to experience significantly higher CXCL chemokine signaling than their aged counterparts (Supplemental Fig. 3A), with chemokine ligands predicted to be produced by a variety of immune and nonimmune cell types including alveolar macrophages, endothelial cells, matrix fibroblasts, and neutrophils themselves. However, these trends were sharply reversed on day 9 post-infection, with aged neutrophils predicted to experience significantly higher CXCL chemokine signaling than young neutrophils in almost all cell-cell interactions (Fig. 3A). Even at this timepoint, there was an interaction predicted between CXCL2 produced by neutrophils and CXCR2 on neutrophils. We quantified *Cxcl2* gene expression by neutrophils using a violin plot and accordingly found that levels of this gene were highest on day 3 post-infection with young neutrophils expressing *Cxcl2* at significantly higher levels than aged neutrophils (Supplemental Fig. 3D). Levels of this gene decreased in both age groups by day 9 post-infection, although less so in aged neutrophils, leading to aged neutrophils expressing significantly higher *Cxcl2* than their young counterparts on day 9 post-infection. Taken together, these data suggest that neutrophils are recruited to the lungs in both young and aged mice early in IAV infection but that they tend to remain in the lungs of aged mice later in the course of infection, potentially allowing them to mediate pathological inflammation. We therefore performed flow cytometry to quantify neutrophils in the lungs of young and aged mice, but found no significant difference in neutrophil frequency on either day 3 or day 9 post-infection (Supplemental Fig. 3E–F). However, we wondered whether the tissue dissociation necessary for flow cytometry may be obscuring important information about neutrophil tissue distribution. We therefore turned to our SPOTlight-deconvoluted Visium data. We classified each capture area of our tissue sections into vascular and parenchymal regions based on the presence of at least 20% vascular cells (endothelial cells, *Car4*^+^ endothelial cells, or *Vwf*^+^ endothelial cells) predicted per capture spot (Fig. 3D). We then compared the distribution of neutrophils between vascular and parenchymal regions on day 9 post-infection and found that neutrophils in aged lungs were significantly more likely to appear in parenchymal regions compared to neutrophils in young lungs (Fig. 3E), suggesting that aging promotes increased parenchymal tissue infiltration by neutrophils, which may then drive lung damage, especially later in the infection course. In support of this hypothesis, previous experiments have shown that antibody-mediated ablation of neutrophils at day 6 post-IAV infection reduces mortality in aged mice, whereas depleting these cells early in infection worsens mortality^8^.

### Lymphocytes from aged mice exhibit characteristics of altered effector function and memory formation

**Fig. 4:**
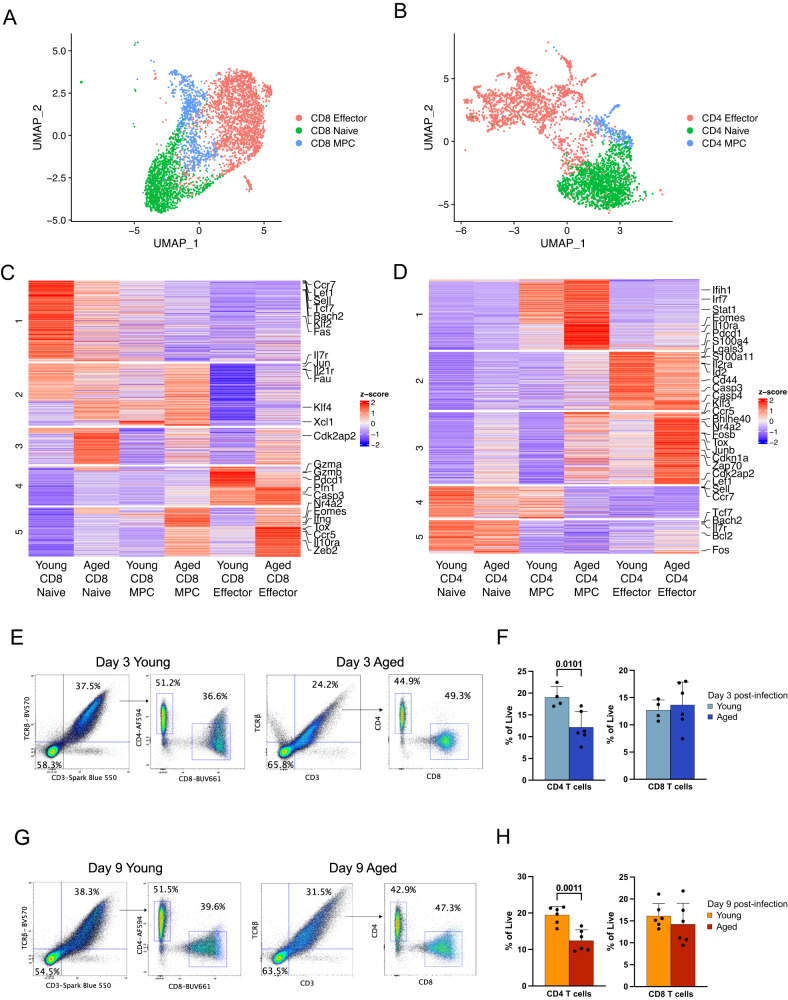
Lymphocytes from aged mice exhibit characteristics of altered effector function and memory formation. **A**, **B** UMAP plots of CD8 (**A**) and CD4 (**B**) T cells from day 3 and day 9 post-infection. **C**, **D** Heatmaps showing differentially expressed genes of CD8 (**C**) and CD4 (**D**) T cell clusters grouped by age. **E**, **F** Representative flow plots (**E**) and quantification (**F**) of CD4 and CD8 T cell frequency on day 3 post-infection. **G**, **H** Representative flow plots (**G**) and quantification (**H**) of CD4 and CD8 T cell frequency on day 3 post-infection. Error bars denote mean ± standard deviation. Statistical testing was performed using a two-sided two-sample *t*-test. Flow cytometry data are pooled from two independent experiments. In (**F**), *n* = 2 young mice per experiment; otherwise, *n* = 3 mice per age group per experiment. Source data are provided as a Source Data file.

It is well established that aging impacts the adaptive immune system, most notably due to a reduction in the generation of naïve T and B lymphocytes^38,39^. We began by separately clustering CD4 and CD8 T cells pooled from all timepoints to identify more granular lymphocyte populations (Fig. 4A, B). By doing so, we found populations of naïve cells, effector cells, and memory precursor cells (MPCs) in both the CD8 and CD4 T cell compartments based on key transcriptional markers (Fig. 4C, D). In particular, we defined naive T cells based on low expression of *Cd44*, MPC T cells on high expression of *Cd44* and *Il7r*, and effector T cells on high expression of *Cd44* and effector genes such as *Bhlhe40* and *Gzmb* (Fig. 4C, D). Although many key marker genes were expressed uniquely in each cluster, we noticed stark differences in expression of key T cell genes by young and aged T cells.

In particular, aged naive CD8 T cells had higher expression of certain markers of naive and memory-like cells such as *Il7r* but lower expression of other such markers, including *Bach2* and *Tcf7* (encodes TCF-1), compared to young naive CD8 T cells (Fig. 4C). Similar differences were observed in young and aged MPC CD8 T cells, likely a result of the similar genetic and epigenetic characteristics of naive, MPC, and memory CD8 T cells^40^. However, there were more unique changes in the gene expression profiles of young and aged effector CD8 T cells. Young effector CD8 T cells had higher expression of genes encoding cytolytic proteins, such as *Gzma* and *Gzmb* (encode granzymes). Conversely, aged effector CD8 T cells had higher expression of genes related to persistent T cell stimulation, T cell exhaustion, and apoptosis, including *Tox*, *Eomes*, and *Casp3* (encodes caspase 3). Interestingly, many of these genes were also upregulated in aged MPC and naive CD8 T cells relative to their young counterparts, although to a lesser degree than observed in aged effector CD8 T cells (Fig. 4C).

Similarly, CD4 T cell subsets also appeared to have differential gene expression based on age (Fig. 4D). Like aged effector CD8 T cells, aged effector CD4 T cells also had higher expression of genes involved in persistent T cell activation, exhaustion, and apoptosis, including *Tox* and *Casp4* (encodes caspase 4), compared to young effector CD4 T cells. Related genes, such as *Pdcd1* and *Nr4a2*, were also expressed at higher levels by aged compared to young MPC CD4 T cells. Aged MPC CD4 T cells also had much higher expression of genes downstream of type I interferon (IFN) signaling, such as *Stat1* and *Irf7*. Interestingly, we recently demonstrated that type I IFN signaling is a key signature of chronic viral infections compared to acute viral infections in both CD4 T cells^41^ and CD8 T cells^42^. Indeed, several markers of T cell exhaustion were upregulated in aged MPC and effector CD4 and CD8 T cells compared to their young counterparts, suggesting that aging causes dysregulation of key cytolytic and memory functions in both CD4 and CD8 T cells.

To determine whether these alterations in gene expression were accompanied by changes in cellular frequencies, we used flow cytometry to quantify CD4 and CD8 T cells in young and aged mice. On day 3 post-IAV infection, we observed no differences in the frequency of CD8 T cells between young and aged mice, but we did observe a significantly reduced frequency of CD4 T cells in aged mice versus young mice (Fig. 4E, F). We found the same pattern at day 9 post-infection, with only aged CD4 T cells, but not CD8 T cells, having reduced frequencies compared to young cells (Fig. 4G, H). This agrees with previously published data demonstrating that aged mice mount a delayed CD4 T cell response to influenza infection compared to young mice^43^. We found that this change appears to be due to a selective reduction in the frequency of naive CD4 T cells in aged mice (Supplemental Fig. 4A–D), in line with known reductions in naive T cell numbers with age as a result of thymic involution^44^. Interestingly, we observed that activated (CD44^+^) CD4 T cells were significantly increased in frequency in aged mice compared to young mice on day 3 but not day 9 post-infection (Supplemental Fig. 4B, D).

We also examined B lymphocytes as antibody responses are critical for anti-IAV immune responses^45^. Using flow cytometry, we observed no differences in the frequency of B cells between young and aged mice on day 3 post-IAV infection (Supplemental Fig. 4E) but did notice a significant increase in the frequency of B cells in aged mice on day 9 post-infection (Supplemental Fig. 4E). We then used our scRNA-seq data to identify genes differentially expressed by age group and time in both naïve B cells (Supplemental Fig. 4I) and plasma cells (Supplemental Fig. 4J). Of note, *Prdm1* (encodes Blimp1), which enables plasma cell antibody secretion^46^, was upregulated in aged mice compared to young mice at all three timepoints studied in both naïve B cells and plasma cells. No significant differences between young and aged mice were found at any timepoint in the expression of *Irf4*^47–49^ or *Xbp1*^50^, both of which are required for plasma cell differentiation. Conversely, *Bach2*, which encodes a basic region-leucine zipper (bZip) transcription factor (TF) that prevents plasma cell formation from naïve B cells by inhibiting the expression of Blimp1^51^, was found to be significantly upregulated in young plasma cells on day 3 post-infection (Supplemental Fig. 4J). In addition, we found that *Pax5*, a master regulator of B cell development whose expression is maintained in memory B cells but lost in plasma cells^52–54^, was upregulated in young naïve B cells at day 3 and day 9 post-infection and in young plasma cells at day 3 post-infection (Supplemental Fig. 4I-J). Given that activated B cells ultimately undergo a cell fate decision by differentiating into either plasma cells or memory B cells^55^, these altered patterns of gene expression suggested that aging may impact the propensity of B cells to develop into plasma cells or memory B cells. We therefore plotted module scores of a gene set upregulated in plasma cells versus memory B cells (GSEA systematic name M3253)^56^ and found that it was significantly upregulated in aged naïve B cells and plasma cells compared to their young counterparts at all time points (Supplemental Fig. 4K), suggesting that aged B cells may be biased to differentiate into plasma cells rather than memory B cells.

Finally, our analyses also emphasized the possibility of aging impacting the function of genes and proteins that have been previously studied without considering age as a variable. For example, we noticed that aged plasma cells upregulated *Ptpn22* (Supplemental Fig. 4J), which encodes Lyp, a phosphatase that inhibits TCR and BCR signaling and is able to impose B cell tolerance in autoimmune disorders such as type 1 diabetes^57–59^. Animal studies conducted on this critical mediator of immune responses have predominantly used relatively young mice (<12 weeks of age), as is standard for most experiments. However, our data suggest that it may be prudent to examine the impact of aging on the function of Lyp and other immunomodulatory proteins. Our data collectively provide insights into aging-induced changes in T and B lymphocytes following influenza infection and may therefore be a useful resource for identifying new pathways in these cells to target for further study.

### Aged endothelial cells exhibit altered localization to fibrotic sites and increased coagulation

Our initial scRNA-seq analysis identified three populations of non-lymphatic endothelial cells in the lung: conventional endothelial cells (ECs), which likely comprise microvasculature; *Vwf*^+^ ECs, which are likely macrovascular cells;^17^ and *Car4*^+^ ECs, which were recently identified as being important for vascular regeneration following lung injury^17,18^. We chose to focus on *Car4*^+^ ECs, as these as are a novel population with direct relevance to lung injury, and conventional ECs, as vascular injury during respiratory viral infection typically affects capillaries rather than large vessels^60^. Although the initial studies identifying *Car4*^+^ ECs used scRNA-seq to categorize this population, they were performed in young mice; we therefore began our analysis by identifying genes differentially expressed between conventional and *Car4*^+^ ECs at different timepoints post-infection and in young and aged mice (Supplemental Fig. 5A). As expected, *Car4*^+^ ECs had unique markers compared to conventional ECs such as *Car4*, *Ednrb* (encodes endothelin receptor B), and *Kdr* (encodes VEGFR-2). In addition, *Car4*^+^ ECs expressed *Cd34* and *Tbx2*, which is expressed by stem-like mesenchymal progenitor cells but downregulated in terminally differentiated endothelial cells^61^, underlining the stem-like nature of *Car4*^+^ ECs. Interestingly, these two genes were expressed at lower levels in *Car4*^+^ ECs at homeostasis but were upregulated following infection, whereas the related T-box TF *Tbx3* was consistently expressed at high levels at all time points (Supplemental Fig. 5A). Conversely, conventional ECs expressed *Cxcl12*, which may serve to recruit monocytes to sites of inflammation via CXCR4-mediated chemotaxis^62^, and *Kit* (encodes c-Kit), which is expressed on progenitor-like ECs recruited to sites of endothelial inflammation for vascular regeneration^63^.

**Fig. 5:**
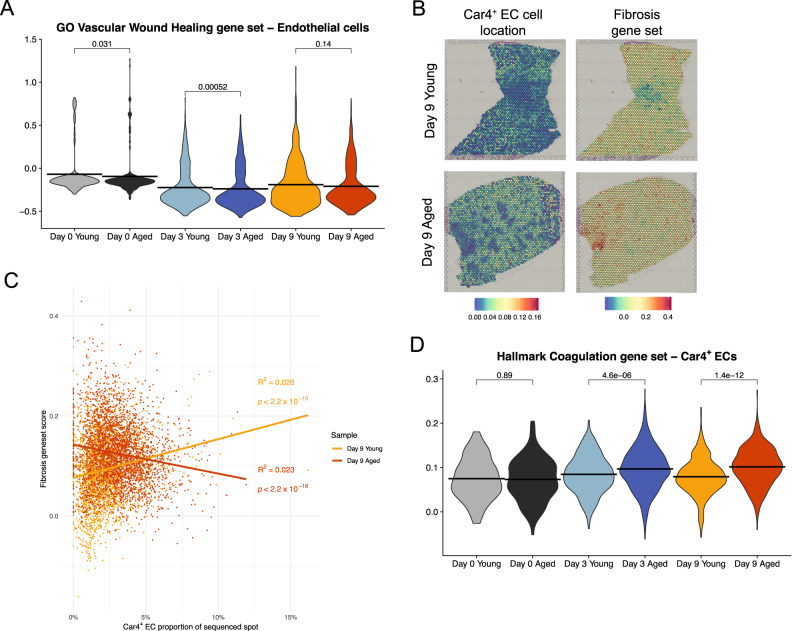
Aged endothelial cells exhibit altered localization to fibrotic sites and increased coagulation. **A** Violin plot showing module scores of a vascular wound healing gene set (GSEA systematic name M29158) in endothelial cells (ECs). Horizontal lines denote mean values. **B** Spatial feature plots showing putative locations of *Car4*^+^ ECs predicted by SPOTlight (left) and fibrosis module scores (right). *Car4*^+^ EC plot color denotes proportion of cells in a capture spot predicted to be *Car4*^+^ ECs. **C** Linear regression between *Car4*^+^ ECs proportion of a capture spot and fibrosis module scores from young and aged lungs day 9 post-infection, as shown in (**B**). Each point denotes one capture spot, color denotes sample identity. **D** Violin plot showing module scores of the Hallmark Coagulation gene set (GSEA systematic name M5946) in *Car4*^+^ ECs. Horizontal lines denote mean values. Statistical testing was performed using a two-sided Wilcoxon test without multiple comparison correction (**A**–**D**) or two-sided *t*-test without multiple comparison correction (**C**).

Given differential expression in terms of genes regulating vascular regeneration, we applied a module score of the GO Vascular Wound Healing gene set (GSEA systematic name M29158) to conventional and *Car4*^+^ ECs. Interestingly, aged conventional ECs exhibited comparable or significantly reduced vascular wound healing scores at all timepoints (Fig. 5A), whereas there was no significant difference between young and aged *Car4*^+^ ECs at any age (Supplemental Fig. 5B). We then turned to our Visium data to determine if there were spatial differences between young and aged *Car4*^+^ ECs, with a focus on fibrotic regions as these cells have been found to localize to regions of tissue damage^17^. Indeed, spatial sequencing identified a significant positive correlation between *Car4*^+^ EC frequency per capture spot and the fibrosis module score (Supplemental Fig. 2F) in young lungs and a significant negative correlation between these variables in aged lungs at day 9 post-infection (Fig. 5B-C). This observation suggests that localization of *Car4*^+^ ECs to sites of tissue damage may be impaired in aged mice.

Our analyses have previously shown increased inflammation in the lungs of aged mice compared to young mice (Fig. 2C, Supplemental Fig. 2E); therefore, it was unsurprising that aged *Car4*^+^ ECs significantly upregulated *Gdf15*, a TGF-β family member and marker of inflammatory stress in lung damage and other pathological states^64^, post-infection compared to both young *Car4*^+^ ECs and conventional ECs (Supplemental Fig. 5A, C). More broadly, aged *Car4*^+^ ECs had significantly higher module scores of the Hallmark Inflammatory Response gene set (GSEA systematic name M5932) than young *Car4*^+^ ECs on day 9 post-infection (Supplemental Fig. 5D). As inflammation is a key trigger of endothelial cell activation and coagulation^65^, we calculated module scores of the Hallmark Coagulation gene set (GSEA systematic name M5946) in ECs (Supplemental Fig. 5E) and *Car4*^+^ ECs (Fig. 5D). Neither cell type exhibited an age-related difference pre-infection, but both aged ECs and aged *Car4*^+^ ECs had significantly higher coagulation module scores at day 3 and day 9 post-infection than their respective young counterparts. Thus, scRNA-seq and spatial sequencing analyses collectively suggested that *Car4*^+^ ECs have impaired localization to sites of tissue damage with age and that aged lungs are prone to endothelial injury, which may lead to increased risk of thrombosis with age during respiratory viral infection. These findings are particularly of interest in light of the COVID-19 pandemic, as SARS-CoV-2 infection is prone to cause more severe infection in the elderly and has been associated with the development of pulmonary thrombosis^66^.

### Spatial sequencing reveals pro- and anti-fibrotic genes differentially expressed by immune and nonimmune cells

**Fig. 6:**
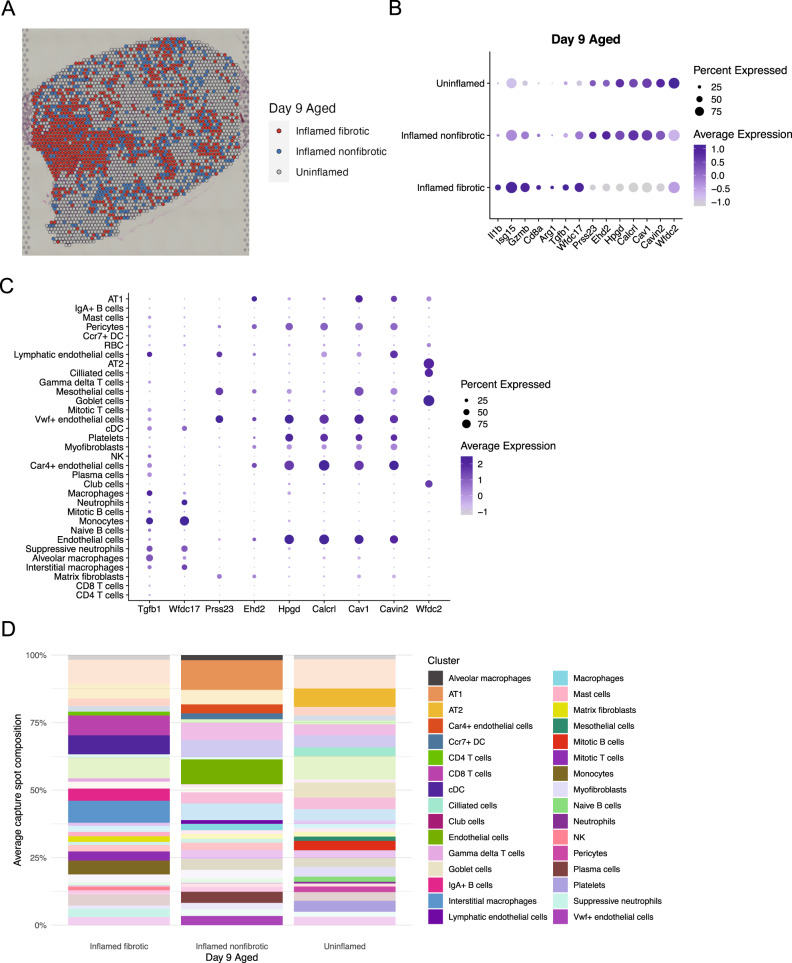
Spatial sequencing reveals pro- and anti-fibrotic genes differentially expressed by immune and nonimmune cells. **A** Spatial feature plot of aged lung tissue from day 9 post-infection. Colors denote inflammation and fibrosis state as determined by capture spot inflammation and fibrosis module scores binarized relative to the mean. **B** Dot plot from day 9 aged lung spatial sequencing data of genes differentially expressed among regions in (**A**). Color denotes expression level, dot size denotes percentage of capture spots in each sample expressing a given gene. **C** Dot plot from scRNA-seq data showing cell type-specific expression of genes in (**B**). Color denotes expression level, dot size denotes percentage of cells expressing a given gene. **D** Bar plot showing average cellular composition of capture spots from each of the three region types in (**A**). Color denotes cell type. A fully opaque bar denotes that a cell type is most enriched in the given region type. See also Supplemental Table 2.

As we found a novel spatial correlation between *Car4*^+^ ECs and tissue fibrosis (Fig. 5B, C), we chose to further investigate the spatial relationship between pulmonary inflammation and fibrosis using their respective module scores (Fig. 2B, D). Using the mean value of each module score as a binary cutoff, we grouped spatial capture spots from our day 9 aged lung sample, which had the highest average fibrosis module score (Supplemental Fig. 2F), into three categories: uninflamed, inflamed fibrotic, and inflamed nonfibrotic (Fig. 6A). We then performed differential expression analysis to identify genes upregulated in each category (Fig. 6B). Some genes, such as *Il1b*, *Isg15* (encodes interferon-stimulated gene 15), *Gzmb* (encodes granzyme b), *Cd8a*, and *Tgfb1* (encodes TGF-β1) were expectedly enriched in inflamed fibrotic regions as these genes encode markers of inflammation and T cell-mediated cytolysis as well as a pro-fibrotic cytokine.

Inflamed fibrotic regions also upregulated *Wfdc17* (encodes AMWAP), which has previously been shown to skew myeloid cells from a pro-inflammatory to a suppressive phenotype characterized by increased *Arg1* (encodes arginase) expression;^67^ in accordance with these data, we also found that *Arg1* was significantly enriched in inflamed fibrotic regions (Fig. 6B) and that *Wfdc17* was selectively expressed by myeloid cells in our scRNA-seq data (Fig. 6C). Furthermore, *Wfdc17* expression tended to be significantly enriched in myeloid cells from aged mice, although expression kinetics varied by specific cell type (Supplemental Fig. 6A–D). These analyses suggest that *Wfdc17* is upregulated by myeloid cells in an attempt to reduce inflammation; however, myeloid skewing towards an anti-inflammatory or myeloid-derived suppressor cell (MDSC) phenotype, characterized by arginase expression^68^, may also promote fibrosis^69^, particularly in aged mice.

We also found several genes that were upregulated in inflamed nonfibrotic and uninflamed regions (Fig. 6B). *Prss23* encodes a protease known to cleave collagen and inhibit fibrosis in renal tissue^70^, although its role in lung fibrosis has not yet been investigated. *Hpgd* encodes hydroxyprostaglandin dehydrogenase, an enzyme that degrades proinflammatory prostaglandins (PGEs) such as PGE2. As PGE2 may prevent lung fibrosis by downregulating TGF-β production in IPF, HPGD has been shown to promote fibrosis in bleomycin models of IPF^71,72^. However, the role of PGE2 in influenza infection is still contested^73^, suggesting that further investigation of the roles of PGE2 and HPGD in IAV, particularly in the context of aging, may be warranted. *Calcrl* encodes the calcitonin receptor like-receptor (CRLR) that mediates adrenomedullin signaling, which has been found to reduce bleomycin-induced pulmonary fibrosis^74^. Interestingly, genes related to caveolae formation, such as *Cav1* (encodes caveolin 1), *Cavin2* (encodes SDPR), and *Ehd2* were all enriched in these regions as well (Fig. 6B). Caveolin 1 has been found to prevent bleomycin-induced fibrosis by inhibiting inflammasome activation^75,76^, although the roles of *Cavin2* and *Ehd2* in pulmonary fibrosis are not well-defined. We also found significantly elevated expression of *Wfdc2* (encodes HE4) in uninflamed regions (Fig. 6B); HE4 is a protease inhibitor that non-covalently binds to PRSS23 to inhibit collagen breakdown in models of renal fibrosis^70^. Given the unusual spatial distribution of expression of *Wfdc2*, its role in fibrosis post-IAV infection may be nuanced.

We then examined expression of these genes by cell type using our scRNA-seq data (Fig. 6C). *Prss23* and *Ehd2* were expressed by both endothelial and epithelial cells; *Hpgd* and *Calcrl* primarily by endothelial cells; *Cav1* and *Cavin2* by a mix of endothelial and epithelial cells; and *Wfdc2* by non-fibroblast epithelial cells including AT1, AT2, club, ciliated, and goblet cells. These data suggest that various immune, epithelial, and endothelial cells interact in the lung to either promote or prevent fibrosis following respiratory viral infection. Age-induced dysregulation of these interactions therefore may bias the lungs to a pro-fibrotic state rather than normal wound healing after injury.

Finally, we returned to our spatial sequencing data to identify which cell types may be enriched in fibrotic areas of the lung. To do so, we deconvoluted our aged day 9 lung sample using SPOTlight (Supplemental Fig. 2D) and calculated the average predicted cellular composition of inflamed fibrotic, inflamed nonfibrotic, and uninflamed capture spots (Fig. 6D and Supplemental Table 2). By highlighting which region each cell type was enriched in, we identified several interesting trends that paralleled our differential gene expression analysis. Notably, cells that expressed high amounts of antifibrotic *Prss23* by scRNA-seq, such as lymphatic endothelial cells and mesothelial cells, were predicted to be enriched in inflamed nonfibrotic and uninflamed regions (Fig. 6C, D). The notable exception to this was matrix fibroblasts, which were unsurprisingly enriched in inflamed fibrotic regions as these cells produce collagen to induce fibrosis^30,77^. Similarly, cell types expressing high levels of *Wfdc2*, such as AT2 cells, ciliated cells, club cells, and goblet cells were all predicted to be enriched in uninflamed regions. Conversely, cells with high expression of *Wfdc17*, such as monocytes, conventional dendritic cells (cDCs), and interstitial macrophages, were predicted to be enriched in inflamed fibrotic regions (Fig. 6D). These analyses demonstrate the unique insights afforded by spatial sequencing, particularly in conjunction with scRNA-seq data, for the discovery of novel transcriptomic and cellular mediators of pathophysiological processes following tissue injury, such as during influenza virus infection.

### Bulk RNA sequencing captures global kinetic and age-induced changes in IAV-infected lungs

To complete our analysis of aging-induced changes in the lung following IAV infection, we performed bulk RNA sequencing (bulk RNA-seq) on young and aged lungs from day 0, day 3, and day 9 post-PR8 IAV infection (Fig. 1A). We visualized samples using a principal component analysis (PCA) plot and observed that samples clustered by timepoint and age, with day 9 samples exhibiting more drastic changes than day 3 samples compared to baseline and aged samples exhibiting more heterogeneity than young samples (Supplemental Fig. 7A). To identify more specific changes, we constructed heatmaps of significant differentially expressed genes (DEGs) identified between young and aged mice at each timepoint (Supplemental Fig. 7B–D). Markers of immune cells such as *Ptprc* (encodes CD45), *Pdcd1*, *Ly6c2*, and *Cd14* were enriched in aged lungs at various timepoints. Markers of B cells (*Cd19*), T cells (*Cd3g*), and antigen presentation (*Cd74* and *Cd83*) were elevated in aged lungs at day 3 post-infection. Genes encoding broader markers of inflammation such as complement proteins (*C1qa*, *C1qb*, and *C1d*), acute-phase proteins (*Crp)*, and interferon-stimulated genes (*Ifit1*, *Ifit3*, *Isg15*, and *Isg20*) were also elevated in aged mice at various timepoints. Interestingly, members of the serpin (serine protease inhibitor) family were upregulated in aged mice across all timepoints (Supplemental Fig. 7B–D). Of particular interest were *Serpina1a*, *Serpina1b*, *Serpina1c*, and *Serpina1d*, which encode isoforms of alpha-1-antitrypsin (A1AT), a protease inhibitor which has been found to inhibit the degradation of elastin and collagen by neutrophils in the lung^78^. Elevated levels of serpins, especially A1AT, could therefore impair collagen breakdown in aged lungs, leading to increased pulmonary fibrosis following influenza infection (Fig. 2D and Supplemental Fig. 2F). In line with this, the profibrotic gene *Wfdc17* (Fig. 6B) was also elevated in aged lungs at day 9 post-infection (Supplemental Fig. 7D). Finally, genes related to coagulation factors and platelets (*F2* and *Ppbp*) were elevated in aged mice at baseline or day 9 post-infection.

**Fig. 7:**
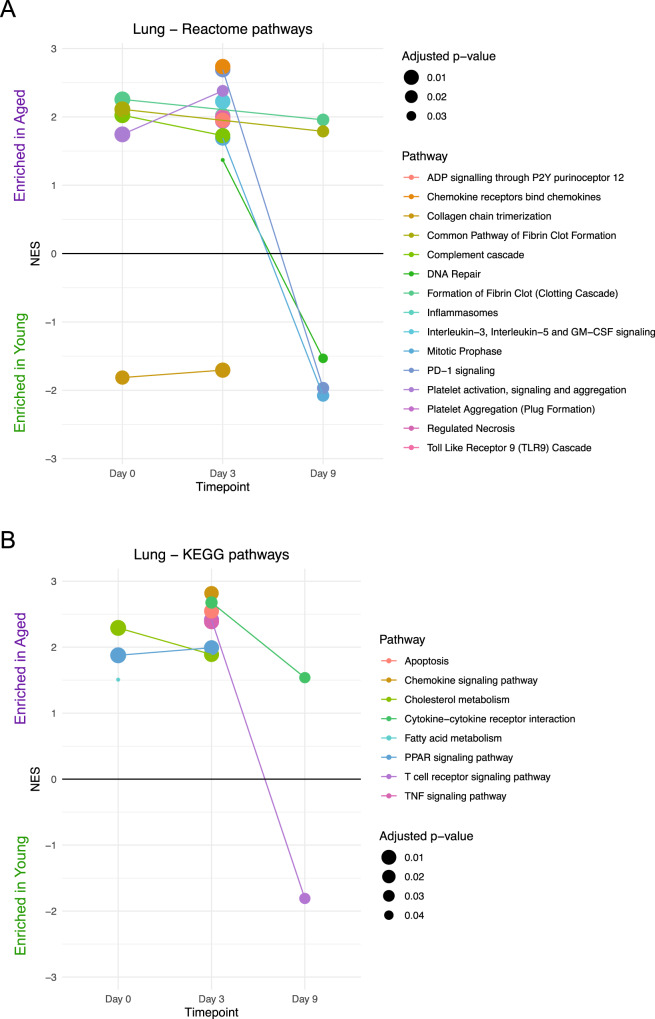
Bulk RNA sequencing captures global kinetic and age-induced changes in IAV-infected lungs. **A** Dot plot showing Reactome pathways enriched in young (normalized enrichment score (NES) < 0) or aged (NES > 0) lungs at day 0, day 3, or day 9 post-infection. Color corresponds to pathway name. Only pathways with adjusted *p*-values < 0.05 are shown. **B** As in (**A**), but with Kyoto Encyclopedia of Genes and Genomes (KEGG) pathways. Statistical testing was performed using a two-sided Kolmogorov-Smirnov test with Benjamini-Hochberg multiple comparison correction.

We then performed gene set enrichment analysis (GSEA) using the Reactome database to identify pathways significantly enriched between young and aged mice at each of the three different timepoints to provide a broad overview of transcriptomic changes in the lung (Fig. 7A). Some pathways, such as collagen chain trimerization were exclusively enriched in young lungs at multiple timepoints. Others were initially enriched in aged lungs but then become enriched in young mice, such as mitotic prophase and DNA repair. Finally, some pathways were consistently enriched in aged lungs including complement cascade; chemokine signaling; necrosis; and several pathways involved in coagulation such as platelet P2Y12 receptor signaling, fibrin clot formation, platelet activation, and platelet aggregation. This supports scRNA-seq module score analyses, which showed elevated coagulation gene sets in multiple endothelial cell populations (Fig. 5D and Supplemental Fig. 5E). We also performed GSEA using the Kyoto Encyclopedia of Genes and Genomes (KEGG) database (Fig. 7B). KEGG results were similar to Reactome results, with apoptosis, chemokine signaling, and cytokine signaling all enriched in aged lungs at different timepoints. Notably, the fatty acid metabolism and PPAR signaling pathways were also enriched in aged lungs. Although global PPARγ expression has been found to be reduced in obese mice post-IAV infection^79^, the role of this signaling pathway requires further investigation in the context of aging and IAV infection, where it may be elevated in aged mice compared to young mice. Collectively, pathway analyses suggest that aged mice may be more prone to pulmonary inflammation, thrombosis, and tissue damage following influenza infection compared to young mice. These detrimental outcomes may be the ultimate result of impaired cellular localization, function, and phenotype due to age-mediated alterations in immune-endothelial-epithelial interactions in the lung following respiratory viral infection.

## Discussion

In this study, we applied a combination of scRNA-seq, spatial sequencing, and bulk RNA-seq to investigate kinetic, spatial, and cell type-specific differences in the host response to influenza infection between young and aged mice. Our analyses revealed drastic changes in gene expression patterns in various immune, endothelial, and epithelial cell types. Moreover, we found that cell-cell interactions, cellular localization, and systemic processes were altered in the lungs by aging. These changes paint a broad picture of increased inflammation, fibrosis, and coagulation as well as reduced vascular wound healing in the aged lung following influenza infection.

By performing scRNA-seq in duplicate on young and aged lungs at 3 and 9 days post-influenza infection, we were able to identify age-induced changes in the host response to infection. In addition, we integrated our data with publicly available scRNA-seq data from lungs of naïve young and aged mice^29^, thus allowing us to compare cellular changes between pre- and post-infection states. This data set by Angelidis et al. also uses young and aged mice on the C57BL/6 genetic background, and tissue collection and dissociation was done in a similar manner to our experimental protocols. Utilizing available data from naïve mice allowed us to make temporal comparisons over the course of infection. Notably, our approach of sequencing all cell types in the lung enabled a broad overview of interactions among various immune, endothelial, and epithelial cells. Our scRNA-seq analysis identified 33 clusters of cells; however, the cellular landscape of the lung is far more heterogeneous than this, and this increased heterogeneity can be identified by either increasing the resolution of the clustering algorithm or selecting subsets of the data and reclustering cells if desired. Our aim in compiling these data is to enable other groups to utilize them to study cell populations of interest, and focusing on specific cells and increasing the cluster granularity may help individual groups with this goal. One limitation to our approach is that frequencies of cells cannot be directly compared between timepoints. Our day 9 samples had to be FACS sorted in order to enrich non-immune populations, thus preventing direct comparisons to day 0 and day 3 samples. However, comparisons can still be made between young and aged mice at each timepoint, as samples from any given timepoint were subject to the same experimental design. Another limitation to our analysis that must be acknowledged pertains to our use of publicly available naïve data^29^ to combine with our scRNA-seq data: the mice in this public data set were 12 and 96 weeks old whereas we used mice that were 16–18 weeks old and 80–82 weeks old. However, these differences in age are relatively minor and would not dramatically impact response to infection. Finally, it must be noted that influenza infection heterogeneously affects different anatomical regions of the lung. Since we consistently sampled the left lung to minimize variability between samples, our spatial transcriptomics data may not fully capture infection heterogeneity in the lungs.

Our spatial sequencing analysis provided a novel perspective on the spatial differences between IAV-infected lungs of young and aged mice. Although the resolution of the 10x Visium platform is currently limited to 55 microns, and is therefore not capable of directly providing single-cell information, we were able to use the SPOTlight algorithm^33^ to deconvolute spatial sequencing data using our scRNA-seq data. Although we chose to use SPOTlight for our analyses, there are multiple software packages available in different programming languages for spatial transcriptomics deconvolution, including cell2location^80^, Stereoscope^81^, and RCTD^82^. We readily identified increased levels of inflammation and fibrosis in the lungs of aged mice using spatial sequencing. Notably, aged mice had significantly elevated levels of inflammation module scores pre-infection. This baseline increase in inflammation, often referred to as inflammaging^34^, is a major driver of age-related pathophysiological changes. Some have argued that inflammaging is not only driven intrinsically by aging but is also accelerated by recurrent bouts of infection and inflammation^83^. This provides one extrinsic mechanism by which aged organisms, which will statistically tend to have a greater history of antigen exposure than young organisms, may have exacerbated dysregulation of the inflammatory response. In line with this model, our spatial sequencing data suggested that aged lungs have a striking correlation between inflamed and fibrotic regions not present in young lungs, further showing that aging impairs the ability to regulate inflammation, which may then cause increased collagen deposition and subsequent fibrosis^14,84^.

When investigating the innate immune system, we used CellChat receptor-ligand analysis and spatial transcriptomics-based location analysis to determine that chemotaxis of neutrophils into the lung parenchyma from the vasculature is likely increased in aged mice compared to young mice at day 9 post-infection, with increased chemokine signaling likely mediating this phenomenon. Previous work has shown that neutrophil infiltration of the lung during the later stages of IAV infection is selectively detrimental to aged mice and that this process is driven by increased CXCL chemokine production by lung epithelial cells^8^. Our work confirms these observations; identifies previously unconsidered chemokine-receptor interactions, such as CXCL12-CXCR4 and PPBP-CXCR2 interactions, that may mediate this migration; and demonstrates that spatial transcriptomics can be used to identify location-based cellular data that may be lost upon tissue dissociation for scRNA-seq or flow cytometry. Finally, our bulk RNA-seq analysis identified significantly increased expression of *Serpina1a* (encodes alpha-1-antitrypsin, A1AT) and other serpin genes in aged lungs, similar to previous findings that A1AT protein levels are increased in bronchoalveolar lavage (BAL) samples from healthy aged human donors^85^. A1AT inhibits the breakdown of elastin and collagen by neutrophils in the lung^78^, thus increasing fibrosis, so increased levels of A1AT may provide an indirect mechanism for neutrophils in aged hosts to contribute to lung damage post-IAV infection.

Concerning the adaptive immune system, scRNA-seq analysis of lymphocytes revealed increased inflammatory activity in the CD4 T cell compartment and altered memory gene expression in the CD4 T cell, CD8 T cell, and B cell pools. Our data suggest a reduction in memory potential in aged CD4 T cells based on decreased expression of the key memory-associated gene *Ccr7*^86–88^ and identify upregulation of genes associated with persistent T cell stimulation and exhaustion, such as *Tox*^89–93^ and *Pdcd1*^94^, in multiple subsets of aged CD4 and CD8 T cells. We and others have shown that the T cell receptor (TCR) sequence and structure, and therefore the make of the T cell repertoire, is important for CD4 T cell fate in the context of acute viral infection^88,95^. However, it has been found that the CD4 T cell repertoire does not appear to contract with age for key influenza antigens^43^. On the other hand, it is known that H-2D^b^:NP_366–374_-specific CD8 T cells contract with age in mice^13^. Human studies have addressed this question at higher resolution by using single-cell T cell receptor sequencing (scTCR-seq) to show that older humans also have reduced antigen-specific CD8 T cells and that the CD8 T cell repertoire of older humans is biased toward private rather than public clonotypes^6,7^. It has also been found that CD8 resident memory T (TRM) cells from young IAV-infected mice have hierarchies of immunodominant antigen specificities in different portions of the respiratory tract^96^. Application of paired spatial sequencing and scTCR-seq to murine models of aging and influenza infection later in the disease course may therefore improve our understanding of how the CD8 T cell repertoire changes with age and how CD8 TRM cells develop post-infection or vaccination, as these cells are critical for prevention of reinfection^96^ but also promote lung fibrosis in aged mice^14^.

Our analysis of B cells suggests that B cells in aged mice may have reduced memory potential and an increased plasmablast or plasma cell phenotype due to decreased expression of the plasma cell master regulator *Prdm1* (encodes Blimp1)^46^ and reduced expression of *Pax5*, a B cell lineage regulating transcription factor whose expression is maintained in memory B cells but lost in plasma cells^52–54^. These observations align with human data showing a loss of memory B cells with age^97,98^ and alterations in memory B cell^99–101^ and plasma cell^102^ functionality following influenza vaccination in aged human subjects, including increased frequencies of PAX5^lo^ atypical B cells^103^ and reduced protective antibody titers^104^. As antibody production is the first line of immune memory against recurrent infections or infections post-vaccination, studies of how influenza-specific B cell dynamics change with age may ultimately help improve influenza vaccine efficacy in the elderly. Application of single-cell B cell receptor sequencing (scBCR-seq) to young mice has shown that individual low- and high-affinity B cell clones can give rise to both memory and plasma cells;^105^ whether this holds true in aged mice is an unanswered but important question as bulk BCR-seq from humans has shown that elderly subjects have reduced IgH diversity pre- and post-influenza vaccination compared to young subjects^106^.

Aging is known to correlate with an increased hypercoagulable state^107^. Our analysis of endothelial cells (ECs) shows that both conventional capillary ECs and recently discovered *Car4*^+^ ECs^17,18^ exhibit increased transcriptomic profiles of coagulation with age, particularly at day 9 post-infection. Clinical data have shown that patients with severe IAV infection have higher serum levels of D-dimer^108^ and that acute respiratory distress syndrome (ARDS) patients with IAV infection are at higher risk of thrombosis compared to patients with ARDS induced by causes other than IAV infection^109^. Our data combine these two sets of observations and imply that changes in vascular phenotype and function may predispose the elderly to a greater risk of thrombotic events following influenza infection. As thrombosis can also occur following SARS-CoV-2 infection and the outcomes of SARS-CoV-2 infection worsen with age^66,110,111^, further studies investigating age-induced changes in vascular biology and hemostasis following respiratory viral infection may be prudent. Our spatial transcriptomics analyses suggest that *Car4*^+^ ECs may exhibit reduced vascular healing in aged mice due to impaired localization to sites of lung fibrosis. As the *Car4*^+^ EC population was discovered relatively recently, the impact of aging on the regenerative role of these cells in respiratory viral infections requires further study.

Finally, we used spatial transcriptomics to identify novel potential regulators of post-IAV pulmonary fibrosis. *Wfdc17* is enriched in fibrotic regions whereas caveolae-associated genes and *Prss23* are enriched in nonfibrotic regions. *Wfdc17* has been reported to play a role in skewing microglia towards an immunosuppressive phenotype^67^ and may therefore warrant further investigation in the context of pulmonary fibrosis. *Prss23*, by contrast, has an established role as an antifibrotic agent in the context of renal fibrosis but is again understudied in the context of respiratory viral infection^70^. The spatially enriched and cell type-specific expression of these genes may help improve our understanding of the cellular mediators of pulmonary fibrosis in the context of infection and aging. More generally, our identification of these genes serves as a proof of concept as to the ability of spatial transcriptomics to identify novel markers that may not be detected by conventional imaging methods or even scRNA-seq.

Finally, our application of bulk RNA-seq to young and aged lung tissue identified global changes in gene expression as well as signaling pathways and pathophysiological processes. For example, we observed alterations in PPAR signaling pathways in the lungs of aged mice. PPAR family members are key regulators of free fatty acid (FFA) metabolism^112^. Obesity and its resultant increase in serum FFAs are also known to negatively impact morbidity and mortality following influenza^113–115^ and SARS-CoV-2^116^ infection. Aged hosts may also be predisposed to negative outcomes following respiratory viral infection due to metabolic dysfunction, although further studies will be needed to expand upon this observation and tease apart these two comorbidities mechanistically. Nonetheless, our current study provides a multifaceted transcriptomic atlas for public investigation of the impact of aging on influenza infection which may be useful to the broad scientific community for hypothesis generation. Moreover, our work demonstrates the immense power and unique discovery potential of paired scRNA-seq and spatial sequencing.

Our study has inherent limitations, most notably that we primarily analyzed transcript-level changes in cellular function, which may not necessarily correlate to protein-level changes and ultimate phenotypic manifestations of disease. Furthermore, our work was done exclusively in female mice, and there are known sex differences in influenza infection in both humans and mice^117^. Finally, our work dissects the lungs at day 3 and day 9 post-IAV infection to investigate age-related changes during infection, but infection resolution and post-infectious lung damage and immune memory generation occur beyond these timepoints^14^. However, the work presented here is presented as a launching off point for other researchers to utilize these data and develop further experiments to address these limitations. As mentioned before, pairing scTCR-seq or scBCR-seq with spatial sequencing may allow for identification of changes in T cell and B cell clonal dynamics with age during influenza infection. In addition, performing high resolution spatial transcriptomics techniques would enable true single-cell spatial analyses, whereas our use of the Visium platform limits us to 55 micron capture spots and necessitates deconvolution using scRNA-seq data. Finally, new methods have bene developed that allow for spatial measurement of protein expression on a large scale, combining the advantages of fluorescence microscopy and spatial transcriptomics^118^.

## Methods

### Ethics Statement

Research for this manuscript complied with all relevant ethical regulations. Young (16–18-week-old) and aged (80–2-week-old) female C57Bl/6 J mice were purchased from Jackson. All mice were maintained under the guidelines of the Institutional Animal Care and Use Committees (IACUC) of the Medical College of Wisconsin (MCW) (protocol AUA00003003). All animal experiments were performed under the guidelines of the Institutional Biosafety Committee (IBC) at MCW (protocol IBC20130979).

### Influenza infection

Mice were infected with ~50 PFU of influenza A/PR8/34 (NCBI Taxonomy ID 211044) to establish acute infection. Infections were performed by intranasal (i.n.) administration under anesthesia as described before^119^.

### Tissue collection

For scRNA-seq, mice were euthanized and the right ventricle was perfused with 10 mL cold DPBS (Corning). The lung lobes were cut into fine pieces and digested at 37 °C with collagenase IV (1 mg/mL; Worthington) for 40 min. The tissue pieces were further ground and homogenized in a 70 μm cell strainer. After spinning down, red blood cells were removed by addition of ACK lysis buffer (Lonza) for 10 min on ice. Cells were kept in 10% RPMI (Lonza) at all times.

For Visium spatial sequencing, mice were euthanized and lungs were inflated with 500 μL of 50% v/v OCT/PBS (Fisher Healthcare) via an intratracheal catheter. The left lung was excised and the caudal half was placed in a cryomold containing OCT. The mold was frozen in an isopentane (Fisher Scientific) bath chilled by liquid nitrogen.

For bulk RNA-seq, pieces of right lung were harvested after ventricular perfusion and snap frozen on dry ice.

For flow cytometry, mice were euthanized and the right ventricle was perfused with 10 mL cold DPBS (Corning). Lungs were minced into fine pieces and placed into a 50 mL tube containing 20 mL of 1.3 mM EDTA in Hank’s Balanced Salt Solution, which was supplemented with 12 mg/mL HEPES sodium salt, 2.9 mg/mL L-glutamine powder, 0.2x penicillin-streptomycin solution, and 0.05 mg/mL gentamycin sulfate (1x HGPG). After shaking for 30 min at 37 °C, the 20 mL supernatant was carefully removed from the minced lung tissue and 25 mL of 1% FBS in cRPMI supplemented with 1 mM CaCl2, 1 mM MgCl2, 2500 units of collagenase I, and 1x HGPG was added to lung tissue. This was placed on a shaker for 60 min at 37 °C. The residual tissue was homogenized in a 70 μm cell strainer. The suspension was centrifuged for 10 min at 1500 rpm. Supernantant was removed and the cell pellet was suspended in 4 mL of 44% Percoll (in 1% cRPMI), and transferred to a 14 mL round bottom tube. Then, 2.5 mL of 67% Percoll (in PBS) was carefully underlaid, and this was centrifuged for 30 min at 1800 rpm at room temperature with no braking. Cells were pipetted out and red blood cell lysis was then performed.

### Cell sorting

Only day 9 post-infection samples were sorted for scRNA-seq to obtain a roughly equal proportion of immune and non-immune cells for analysis, although all samples were stained to examine the relative proportions of immune and non-immune cells. Single-cell suspensions were incubated with biotin-conjugated antibodies targeting the following Lineage markers for 30 min at 4 °C in 10% RPMI: CD3e, CD8a, TCRb, B220, NK1.1, Ly6C/G (anti-GR1), CD11b, CD11c, Ter119 (Biolegend). Samples were then washed twice in 10% RPMI and stained with Live/Dead Fixable Violet (ThermoFisher), streptavidin, and anti-CD90 for 30 min at 4 °C in 10% RPMI. Samples were then run on an Aria IIIu cell sorter (BD Biosciences).

### Flow cytometry

Live/Dead cell staining was performed using Fixable Live Dead Blue (ThermoFisher) in PBS at 4 °C for 30 min in the dark. Cells were washed twice and surface panel was applied to cells, incubated at room temperature for 60 min in the dark. Cells were fixed using 1x TrueNuclear Fix (Biolegend), and intracellular transcription factor staining was performed using TrueNuclear Perm Buffer (Biolegend) at room temperature for 45 min in the dark. Cells were resuspended in FACS buffer prior to analysis by flow cytometry. Single color compensations and cellular flow cytometry were acquired and unmixed on a 5 laser spectral flow cytometer (Cytek Aurora). Data were analyzed using Cytobank Premium (Beckman Coulter). Antibodies and dilutions are listed in Supplemental Table 3.

### Single-cell RNA sequencing and analysis

For day 9 post-infection samples, single-cell suspensions of lung tissue were FACS sorted (see above), and Lin^+^ cells and Lin^–^ cells from each sample were individually pooled at a 1:1 ratio. For day 3 post-infection samples, cells were not sorted as the ratio of Lin^+^:Lin^–^ cells was ~1:1. Cells were loaded onto the Chromium Controller (10x Genomics), then the 10x Genomics 3’ v2 Reagent Kit (day 9 post-infection samples) or the 10x Genomics 3’ v3.1 Reagent Kit (day 3 post-infection samples) was used to generate cDNA libraries. Barcoded libraries were sequenced on an Illumina NextSeq 500 instrument using a NextSeq 500/550 High Output Kit v2 (150 cycles) (20024907, Illumina) with the following cycle counts: 28 (read 1), 8 (index), and 91 (read 2) (day 9 post-infection samples) or 28 (read 1), 10 (index 1), 10 (index 2), 90 (read 2) (day 3 post-infection samples). Data were demultiplexed and aligned to the mm10 2020-A reference transcriptome (10x Genomics) using Cell Ranger (v6.0, 10x Genomics). Analysis was performed in R using Seurat (v4.0)^120^, with tidyverse (v1.3.1) used for data organization^121^. Quality control was performed to exclude low quality cell, doublets, and dead cells; cells with >1000 unique genes (day 0 samples), >3500 unique genes (day 3 and day 9 samples), <200 unique genes (all samples), or >10% mitochondrial genes were excluded from the final analysis. Day 3 and day 9 post-infection samples collected by our lab were then integrated with publicly available day 0 data^29^ using Seurat integration pipeline. Gene set scoring was performed using Seurat’s AddModuleScore function with default settings for both scRNA-seq and spatial transcriptomics data. Receptor-ligand analysis was performed using CellChat (v1.1.3)^37^ with default settings.

### Bulk RNA sequencing and analysis

Mice were infected and lungs were inflated as described above. Small sections of the right lung were excised and snap frozen on dry ice. RNA was extracted using an RNAEasy Mini Kit (Qiagen). Reverse transcription was performed and the resultant cDNA was enzymatically fragmented and indexed using a Nextera XT DNA Library Preparation Kit (Illumina). Barcoded cDNA libraries were sequenced on an Illumina NextSeq 500 instrument using a NextSeq 500/550 High Output Kit v2 (75 cycles) (20024906, Illumina) with the following cycle counts: 37 (read 1), 8 (index 1), 8 (index 2), 37 (read 2). Demultiplexing was performed using bcl2fastq (v2.20, Illumina). Alignment was performed using Salmon (v1.4.0)^122^ and the GRCm39 reference genome (Ensembl) with default settings. Downstream analysis was performed in R using DESeq2 (v1.28.1)^123^.

### Visium spatial sequencing and analysis

10 μm sections were generated from frozen inflated left lungs using a cryostat and placed on barcoded slides (10x Genomics). The Visium Spatial Gene Expression Slide & Reagent Kit (10x Genomics) was used to generate barcoded cDNA libraries. H&E stained tissues on Visium slides were imaged using a Nikon Eclipse Ti2 inverted microscope. Lung tissue on Visium slides was permeabilized for 14 min to extract mRNA. Barcoded cDNA libraries were sequenced on an Illumina NextSeq 500 instrument using a NextSeq 500/550 High Output Kit v2 (150 cycles) (20024907, Illumina) with the following cycle counts: 28 (read 1), 10 (index 1), 10 (index 2), 90 (read 2). Loupe Browser (v5.0, 10x Genomics) was used to identify which spatial sequencing capture area spots were in contact with tissue. Demultiplexing and alignment was performed with Space Ranger (v2.1, 10x Genomics) and the mm10 2020-A reference transcriptome (10x Genomics). Analysis was mainly performed in R using Seurat (v4.0). Visium datasets were integrated using the SCTransform pipeline, and PCA and UMAP were performed using the top 30 principal components. SPOTlight^33^ was used to demultiplex Visium data with our integrated scRNA-seq data used as a reference.

### Statistical analysis

Statistical comparisons for sequencing data were performed in R. Pairwise comparisons were performed using a Wilcoxon test with Holm-Sidak correction through the ggpubr package. Linear regressions were performed using the lm function. CellChat analyses were performed using default parameters.

### Reporting summary

Further information on research design is available in the Nature Portfolio Reporting Summary linked to this article.

### Supplementary information

Supplementary Information

Reporting Summary

### Source data

Source Data

## Data Availability

Single-cell RNA sequencing, spatial sequencing, and bulk RNA sequencing data from this paper are available in the GEO database with accession numbers GSE202325, GSE202322, and GSE202324, respectively. The flow cytometry data generated in this study are provided in the Source Data file. Source data are provided with this paper.

## Code availability

All code and scripts are available at https://github.com/ryanjbrown21/Flu_Spatial_Sequencing.
