# Supplementary material for: A spatial sequencing atlas of age-induced changes in the lung during influenza infection
Source: Nat Commun. 2023 Oct 18;14:6597. doi: 10.1038/s41467-023-42021-y (PMC10584893; doi:10.1038/s41467-023-42021-y)
Supplement: Supplementary file 1 — Supplementary Information [file 41467_2023_42021_MOESM1_ESM.pdf]

Supplemental Figure 1

A

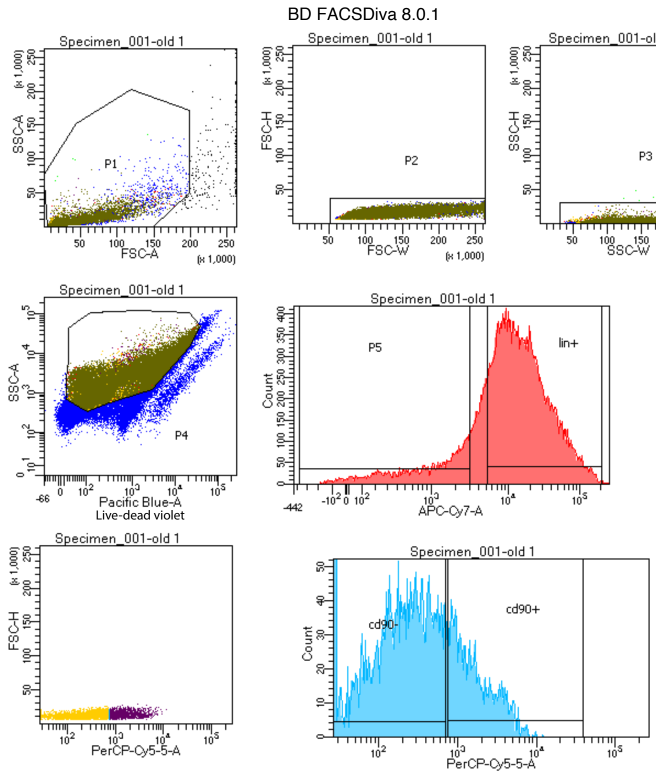

Tube: old 1

Population	#Events	%Parent	%Total
All Events	50,000	####	100.0
P1	49,785	99.6	99.6
P2	49,785	100.0	99.6
P3	49,780	100.0	99.6
P4	26,735	53.7	53.5
P5	4,381	16.4	8.8
cd90+	1,043	23.8	2.1
cd90-	3,283	74.9	6.6
lin+	19,377	72.5	38.8

Global Sheet1

Printed on: Fri Apr 23, 2021 02:00:45 CDT

B

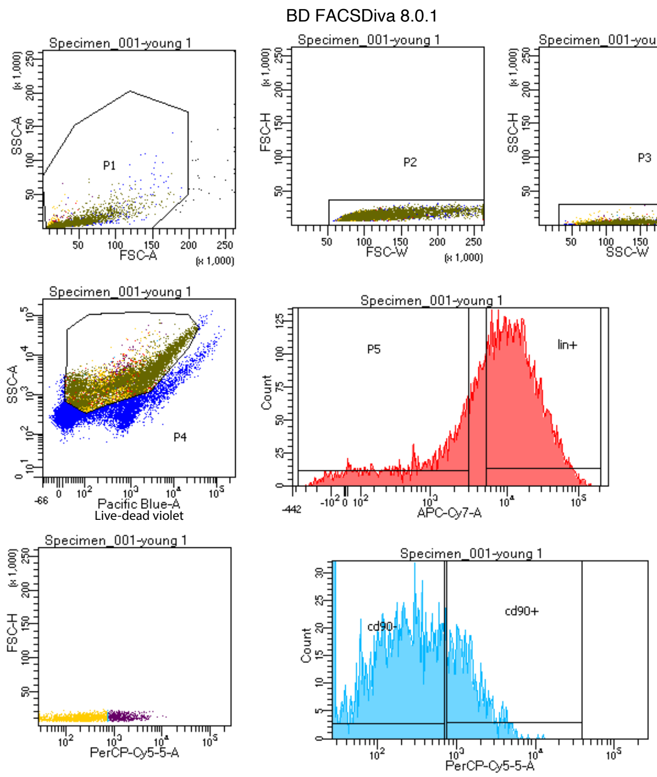

Tube: young 1

Population	#Events	%Parent	%Total
All Events	19,596	####	100.0
P1	19,564	99.8	99.8
P2	19,564	100.0	99.8
P3	19,564	100.0	99.8
P4	9,446	48.3	48.2
P5	2,389	25.3	12.2
cd90+	531	22.2	2.7
cd90-	1,831	76.6	9.3
lin+	5,762	61.0	29.4

Global Sheet1

Printed on: Fri Apr 23, 2021 02:00:46 CDT

Supplemental Figure 1. FACS sorting strategy for scRNA-seq of day 9 post-infection samples.

(A) Representative FACS plot of an aged lung sample. The gates "P5" and "lin+" were sorted and pooled at a 1:1 ratio for sequencing. Lin: CD3, CD8a, TCRβ, TCRγ/δ, CD11b, CD11c, B220, Gr1, NK1.1, and TER119. **(B)** As in (A), but of a young lung sample.

Supplemental Figure 2

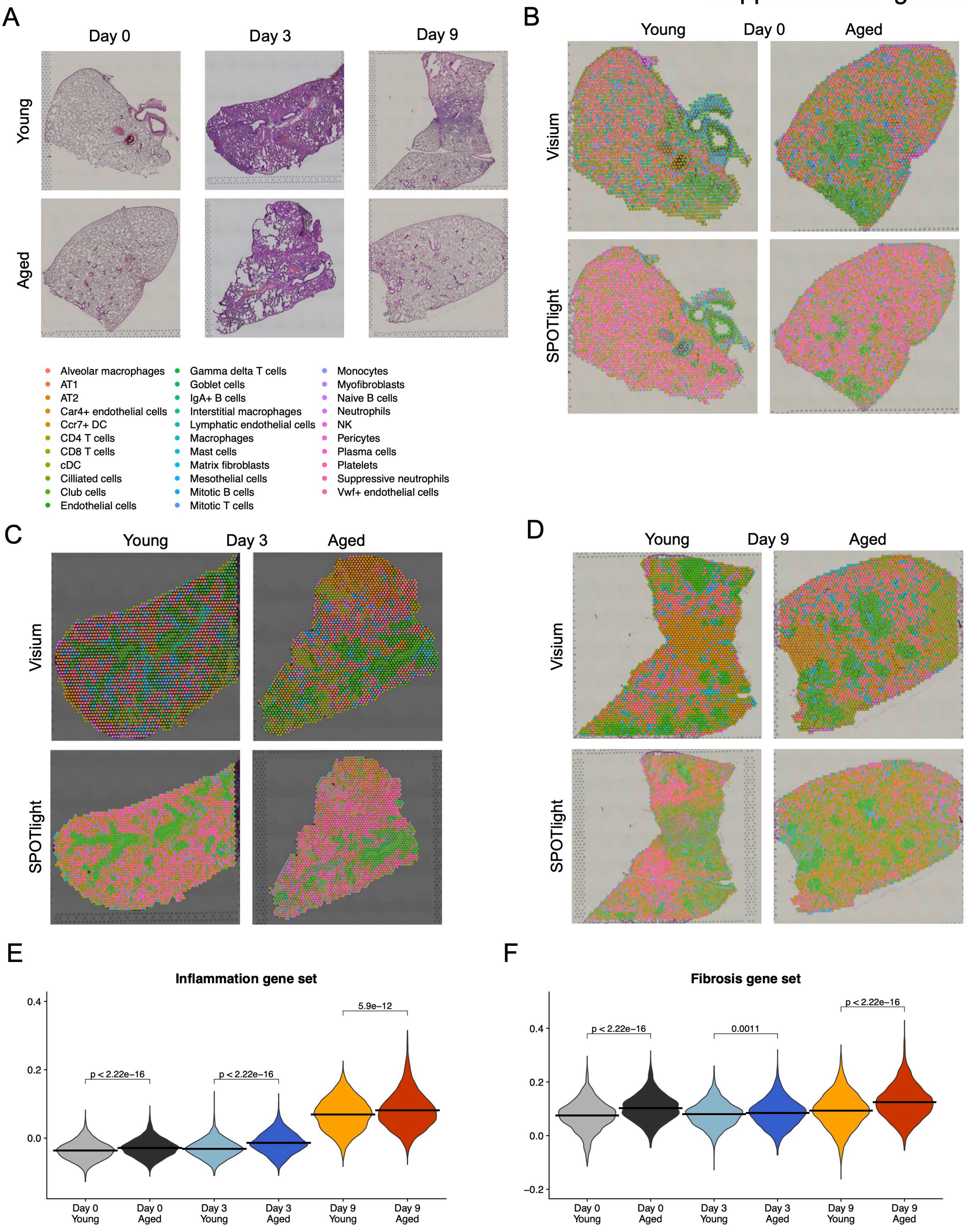

Supplemental Figure 2. Visium spatial sequencing capture spot identities.

(A) Hematoxylin and eosin (H&E) stained left lung sections ultimately used for Visium spatial sequencing. **(B-D)** Capture spot identities superimposed on H&E stained sections for young and aged lung sections from day 0 **(B)**, day 3 **(C)**, and day 9 **(D)** post-infection. Top rows are colored by Seurat UMAP cluster identity (Figure 2A), bottom rows by putative cellular identities assigned by SPOTlight following scRNA-seq based deconvolution. RBCs were excluded from SPOTlight analysis. **(E-F)** Violin plots showing module scores of inflammation **(E)** and fibrosis **(F)** in spatial sequencing data. Horizontal lines denote mean values.

Supplemental Figure 3

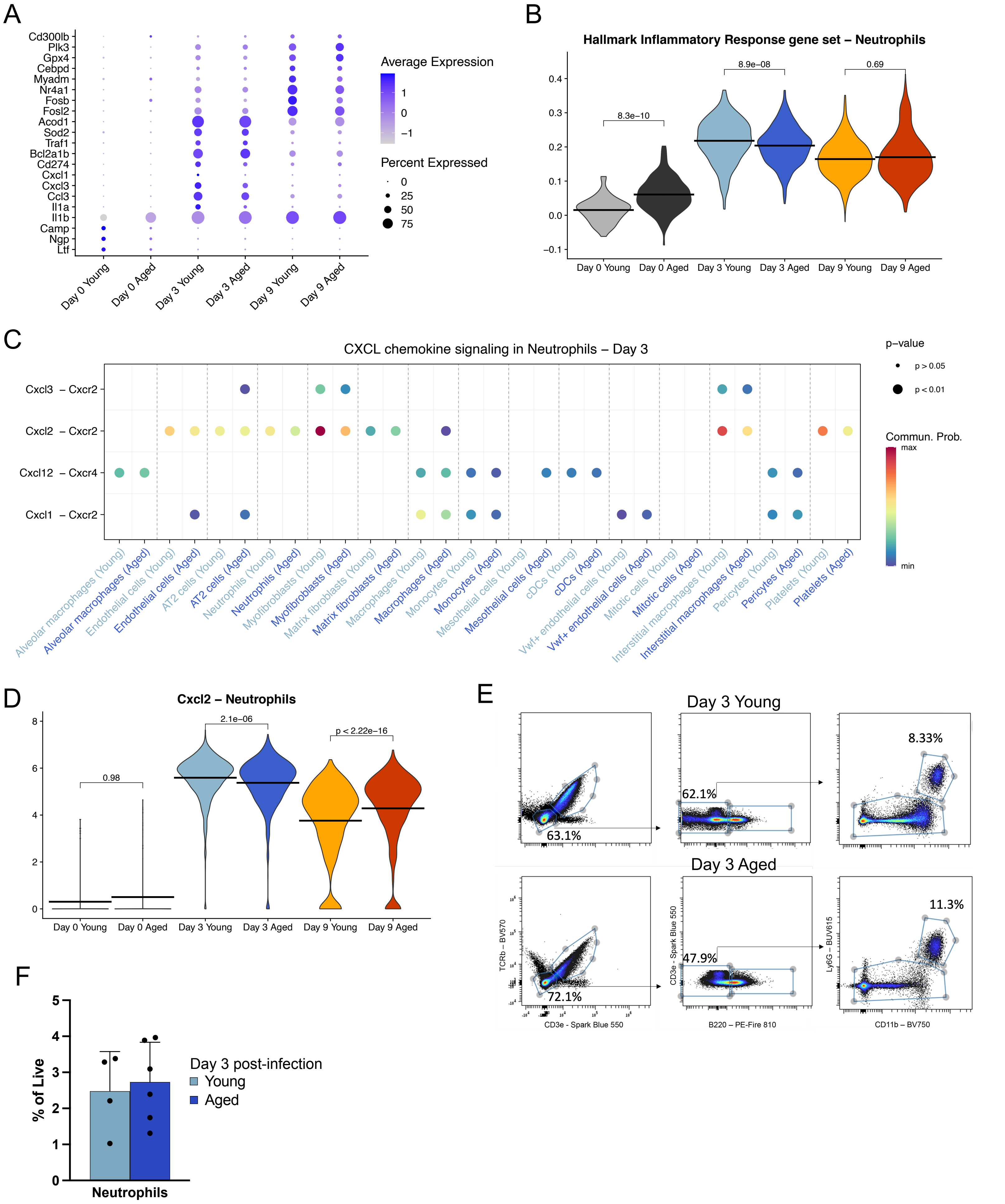

Supplemental Figure 3. Transcriptomic and flow cytometric characterization of young and aged neutrophils.

(A) Dot plot showing genes differentially expressed among neutrophils from different time points and age groups. Color denotes expression level, dot size denotes percentage of cells in each sample expressing a given gene. **(B)** Violin plot showing module scores of the Hallmark Inflammatory Response gene set (GSEA systematic name M5932) in neutrophils from different timepoints and age groups. Horizontal lines denote mean values. **(C)** Dot plot showing predicted interactions between CXC chemokine ligands produced by cells labeled on the x-axis and CXC chemokine receptors located on neutrophils at day 3 post-infection. Color denotes communication probability, size denotes *p*-value. **(D)** Violin plots showing expression of *Cxcl2* in neutrophils. Horizontal lines denote mean values. **(E-F)** Representative flow plots **(E)** and quantification **(F)** of $Cd11b^+$ $Ly6G^+$ neutrophil frequency on day 3 post-infection. Error bars denote mean ± standard deviation. Flow cytometry data are pooled from two independent experiments, n = 2-3 mice per age group per experiment.

Supplemental Figure 4

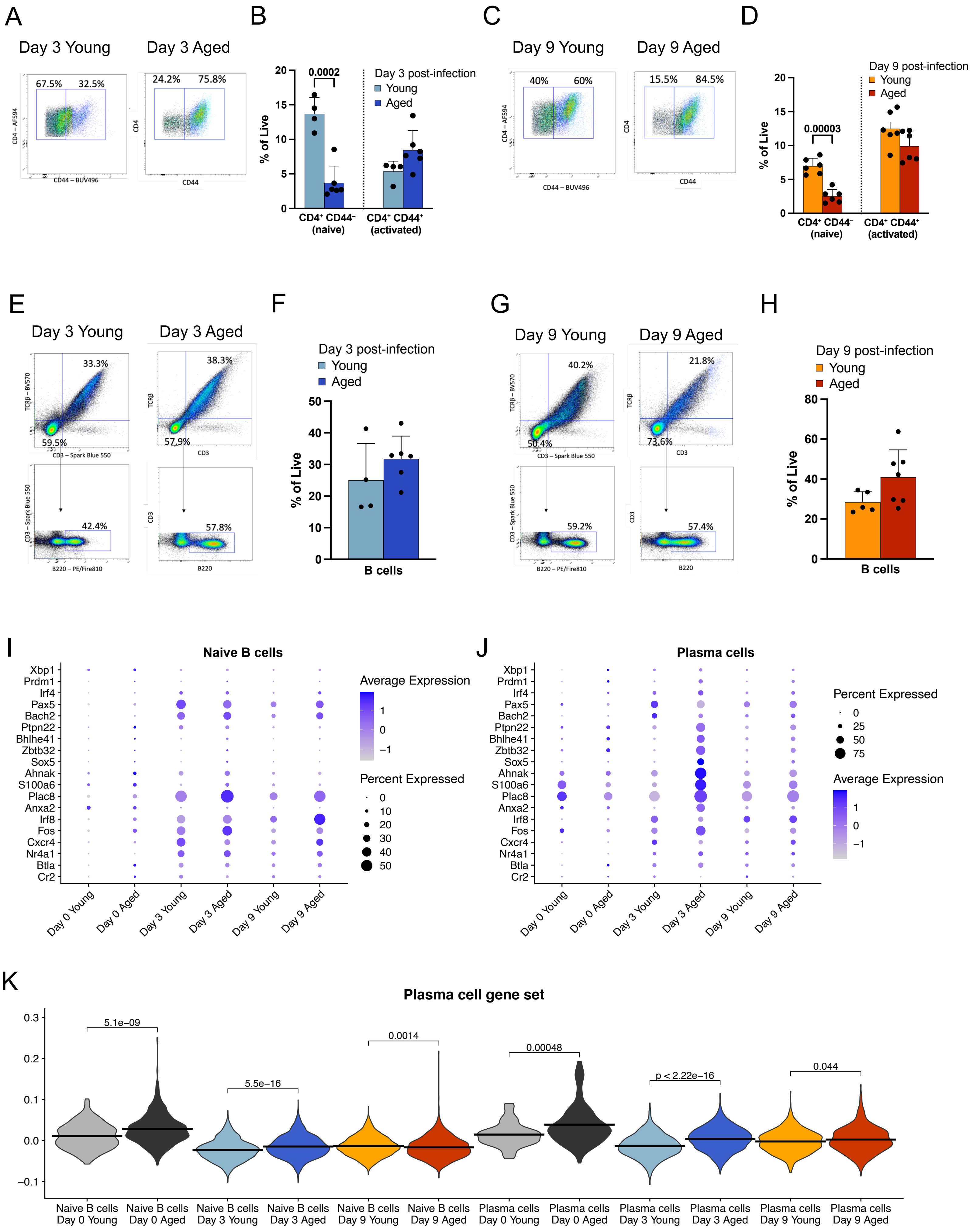

Supplemental Figure 4. Flow cytometric and transcriptomic analyses of young and aged lymphocytes.

(A-B) Representative flow plots **(A)** and quantification **(B)** of $CD44^{-}$ (naïve) and $CD44^{+}$ (activated) CD4 T cell frequency on day 3 post-infection. **(C-D)** As in (A-B), but on day 9 post-infection. **(E-F)** Representative flow plots **(E)** and quantification **(F)** of B cell frequency on day 3 post-infection. **(G-H)** Representative As in (E-F), but on day 9 post-infection. **(I-J)** Dot plot showing expression of differentially expressed genes in naïve B cells **(I)** and plasma cells **(J)**. Color denotes expression level, dot size denotes percentage of cells expressing a given gene. **(K)** Violin plot showing module scores of a gene set upregulated in plasma versus memory B cells (GSEA systematic name M3253) [56], in young and aged naïve B cells and plasma cells. Horizontal lines denote mean values. In (A-H), error bars denote mean ± standard deviation. ***, $p < 0.001$ and ****, $p < 0.0001$ by two-sample *t*-test. Flow cytometry data are pooled from two independent experiments, n = 2-3 mice per age group per experiment.

Supplemental Figure 5

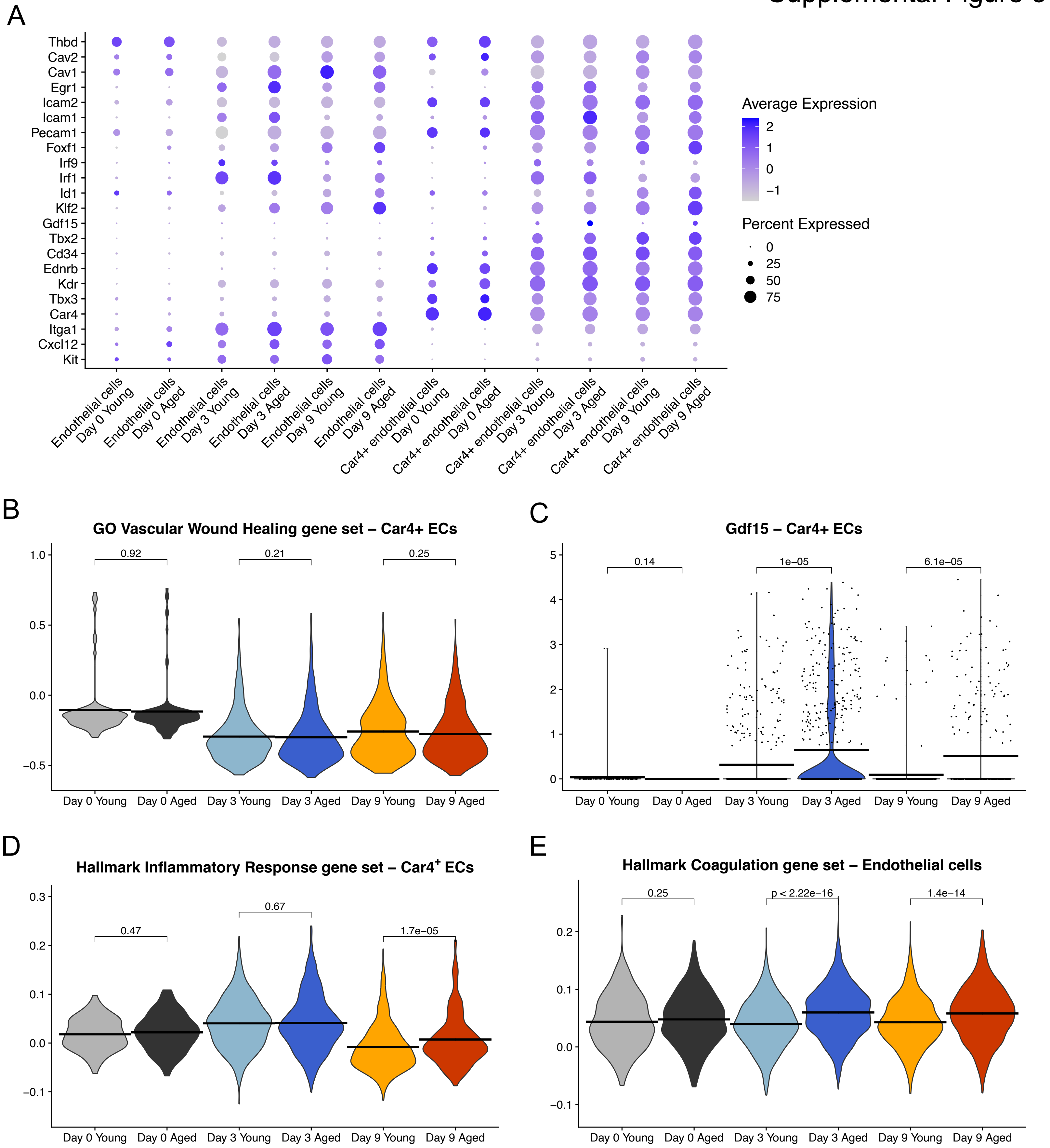

Supplemental Figure 5. Genes differentially expressed among young and aged endothelial cells post-IAV infection.

(A) Dot plot showing expression of differentially expressed genes in endothelial cells (ECs) and *Car4*$^+$ ECs. Color denotes expression level, dot size denotes percentage of cells expressing a given gene. **(B)** Violin plot showing module scores of the GO Vascular Wound Healing gene set (GSEA systematic name M29158) in *Car4*$^+$ ECs. Horizontal lines denote mean values. **(C)** Violin plots showing expression of *Gdf15* in *Car4*$^+$ ECs. Horizontal lines denote mean values. **(D)** Violin plot showing module scores of the Hallmark Inflammatory Response gene set (GSEA systematic name M5932) in *Car4*$^+$ ECs. Horizontal lines denote mean values. **(E)** Violin plot showing module scores of the Hallmark Coagulation gene set (GSEA systematic name M5946) in ECs. Horizontal lines denote mean values.

Supplemental Figure 6

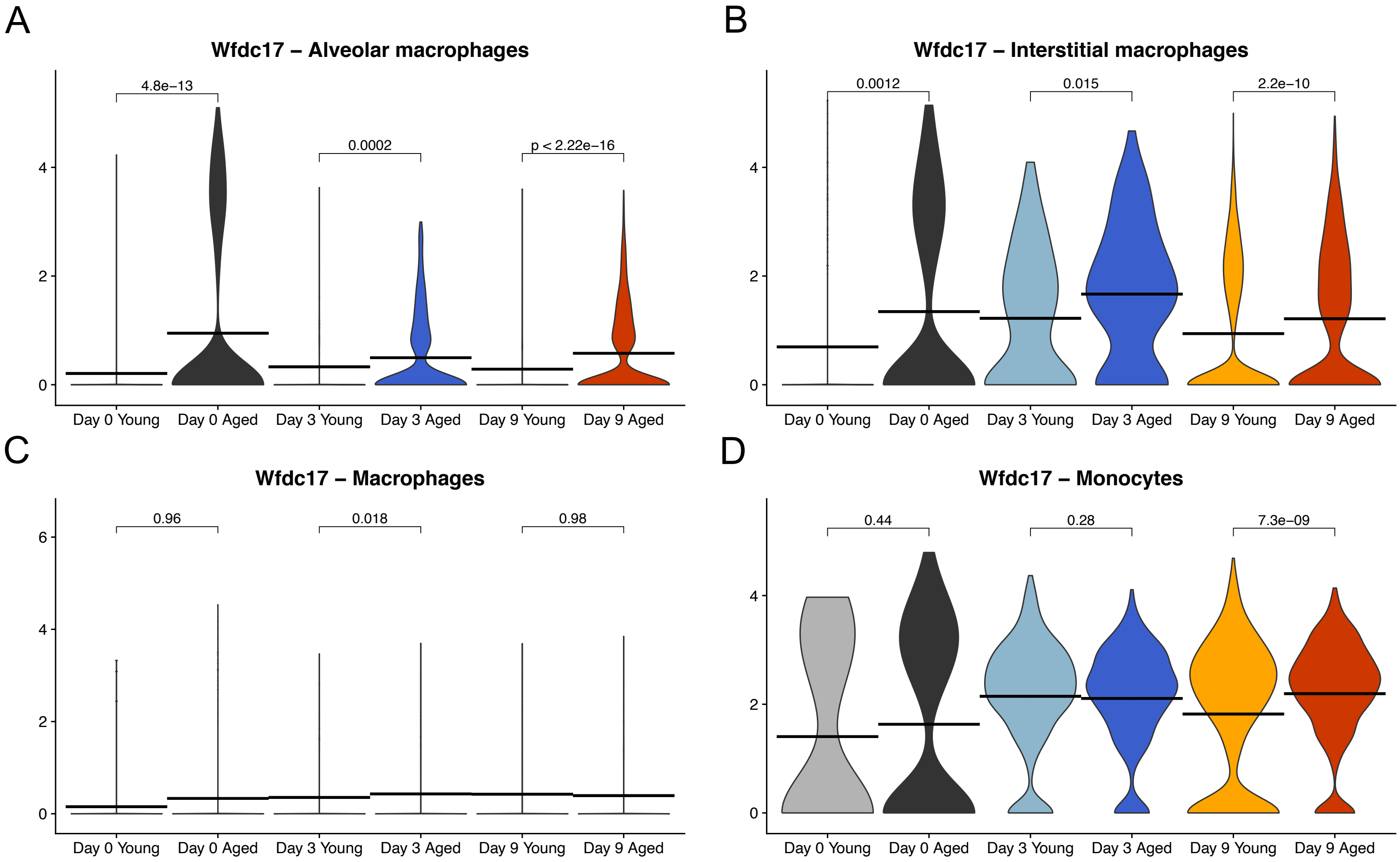

Supplemental Figure 6. Differential expression of inflammation and fibrosis marker genes. **(A-B)** Violin plots showing expression of *Gdf15* in alveolar macrophages **(A)** and interstitial macrophages **(B)** from young and aged mice at multiple timepoints post-infection. Horizontal lines denote mean values. **(C-D)** Violin plots showing expression of *Wfdc17* in macrophages **(C)** and monocytes **(E)** from young and aged mice at multiple timepoints post-infection. Horizontal lines denote mean values.

Supplemental Figure 7

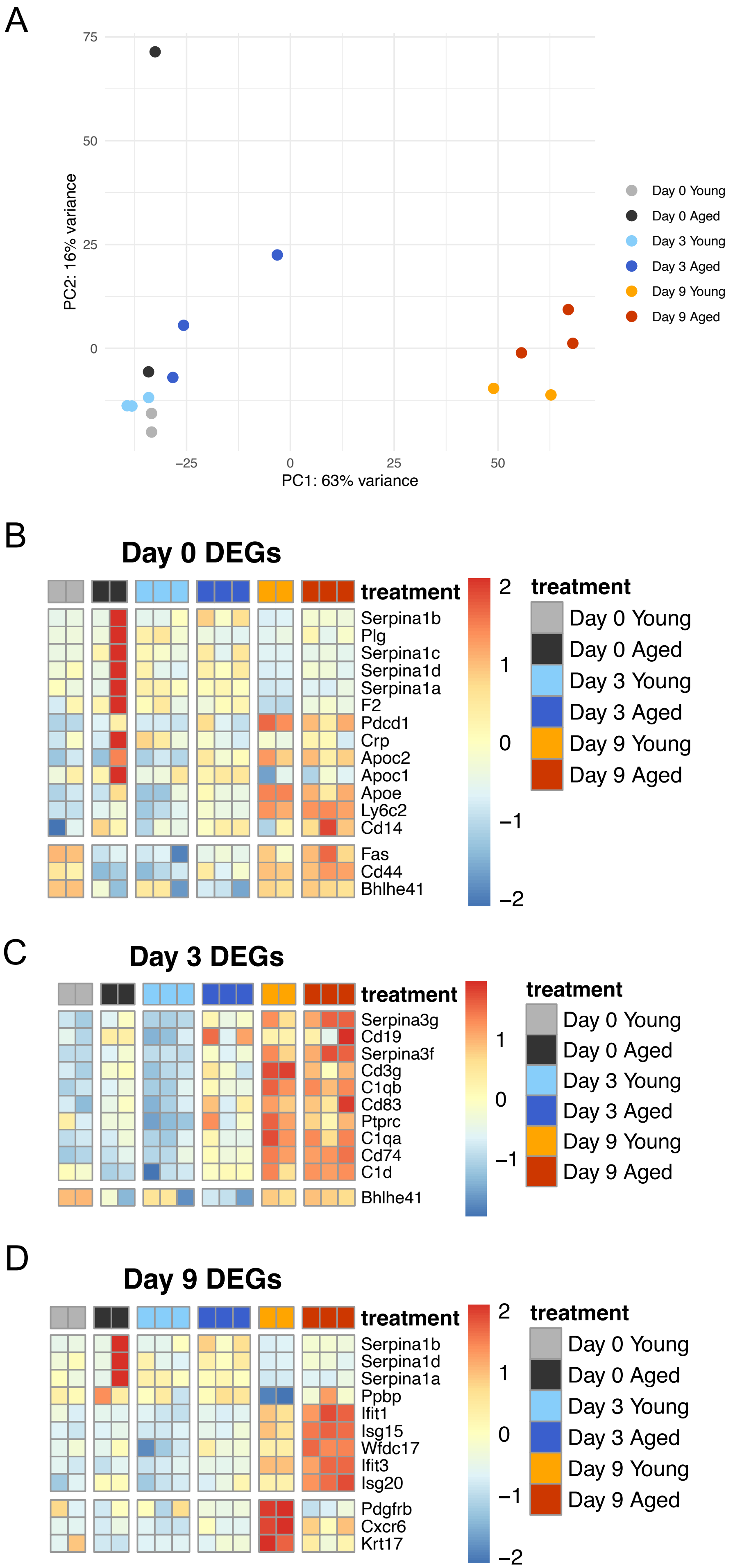

Supplemental Figure 7. Bulk RNA-seq of young and aged lungs at multiple timepoints post-IAV infection.

(A) Principal component analysis (PCA) plot of bulk RNA-seq samples. Each point represents one replicate, colors denote sample identity. **(B-D)** Heatmaps showing scaled expression of selected genes differentially expressed at a significant level (adjusted $p < 0.05$) between young and aged mice at day 0 **(B)**, day 3 **(C)**, and day 9 **(D)** post-infection. Adjusted *p*-values in (B-D) were calculated using the Wald test.

	Cell_type	Young	Aged
1	CD4 T cells	6.803175133	3.55861244
2	CD8 T cells	6.438463922	6.738437002
3	Matrix fibroblasts	3.30623823	3.401614833
4	Interstitial macrophages	8.111844771	4.281299841
5	Alveolar macrophages	9.461037878	5.98335327
6	Suppressive neutrophils	0.421920812	0.712719298
7	Endothelial cells	7.258468213	7.715311005
8	Naive B cells	6.452766323	8.086622807
9	Monocytes	4.850897476	5.078748006
10	Mitotic B cells	6.297823651	8.742025518
11	Neutrophils	4.331243594	4.956638756
12	Macrophages	3.034492622	5.123604466
13	Club cells	5.053514815	2.701355662
14	Plasma cells	2.312221401	6.603867624
15	Car4+ endothelial cells	2.48623394	2.629086922
16	NK	2.512455007	2.028508772
17	Myofibroblasts	3.744845177	4.440789474
18	Platelets	2.264546733	2.065889155
19	cDC	2.03094086	1.981160287
20	Vwf+ endothelial cells	1.444542442	1.871511164
21	Mitotic T cells	1.973731258	1.123903509
22	Goblet cells	1.725822984	1.153807815
23	Mesothelial cells	1.132273367	1.731957735
24	Gamma delta T cells	1.084598698	1.577452153
25	Cilliated cells	0.929656027	1.335725678
26	AT2	1.511286978	0.62300638
27	Lymphatic endothelial cells	0.858144025	0.854764753
28	RBC	0.648375486	0.949461722
29	Ccr7+ DC	0.533956282	0.615530303
30	Pericytes	0.424304546	0.316487241
31	Mast cells	0.307501609	0.406200159
32	IgA+ B cells	0.123954137	0.518341308
33	AT1	0.128721604	0.092204944

Supplemental Table 1. Breakdown of scRNA-seq data by cell type.
Frequency of cellular clusters within scRNA-seq data from each age group (young and aged) were tabulated. Data were pooled from all timepoints (day 0, day 3, and day 9 post-infection) and integrated and analyzed with Seurat. Corresponds to Figure 1D.

	Cell_type	Inflamed_fibrotic	Inflamed_nonfibrotic	Uninflamed
1	Alveolar.macrophages	1.728760987	1.890181217	1.528542561
2	AT1	8.967439709	11.00396754	10.8755701
3	AT2	5.434695078	5.371563972	6.792571351
4	Car4..endothelial.cells	2.756724733	3.341695742	3.185731855
5	Ccr7..DC	2.07572143	2.182527969	1.91109442
6	CD4.T.cells	1.408859411	1.27188322	1.322898648
7	CD8.T.cells	7.358628164	6.411615012	4.125657398
8	cDC	7.041854432	6.304901501	4.442487664
9	Cilliated.cells	1.242116652	0.852734902	3.247152658
10	Club.cells	2.93E-18	2.06E-18	4.29E-19
11	Endothelial.cells	7.637257795	9.183527864	8.658350473
12	Gamma.delta.T.cells	1.217227748	1.185468338	1.15892949
13	Goblet.cells	2.572130397	1.843439473	5.55131059
14	IgA..B.cells	4.511638961	4.120846881	4.274699391
15	Interstitial.macrophages	8.090469226	6.156160807	4.162938092
16	Lymphatic.endothelial.cells	1.267086358	1.373136431	1.307336464
17	Macrophages	2.170927816	2.363241683	1.678075782
18	Mast.cells	1.618328417	1.364041814	1.219943681
19	Matrix.fibroblasts	2.080714101	1.858161068	1.780126686
20	Mesothelial.cells	1.174520248	1.451300748	1.589338542
21	Mitotic.B.cells	2.382655569	2.690646715	3.495908362
22	Mitotic.T.cells	3.359484187	3.26134006	2.758789042
23	Monocytes	5.098790445	4.047585355	3.385684424
24	Myofibroblasts	3.009926005	3.177534962	3.59416251
25	Naive.B.cells	0.974842405	1.57996478	1.912884428
26	Neutrophils	0.60394296	0.467272345	0.648908933
27	NK	1.344168364	1.134494588	1.087933991
28	Pericytes	1.602152182	1.797663655	2.081369031
29	Plasma.cells	4.029898139	4.052396012	3.210852372
30	Platelets	1.156008369	2.395787361	4.056451402
31	Suppressive.neutrophils	3.058998848	2.494405596	1.85130545
32	Vwf..endothelial.cells	3.024030863	3.370512388	3.102994211

Supplemental Table 2. Predicted composition of fibrotic spatial capture spots.
Predicted cellular composition of Visium spatial transcriptomic capture spots previously categorized as inflamed fibrotic, inflamed non-fibrotic, and uninflamed (see Figure 6A). Visium sections were analyzed with Seurat and cellular composition was predicted using SPOTlight. Data are from aged lung tissue on day 9 post-infection.
Corresponds to Figure 6D.

Color	Marker	Clone	Manufacturer	Dilution
AF594	CD4	GK1.5	Biolegend	1:200
AF647	TCR gamma delta	GL3	Biolegend	1:200
APC	H2Db PA tetramer	–	NIH	1:100
APC-Cy7	SiglecF	E50-2440	BD	1:200
BUV496	CD44	IM7	BD	1:200
BUV615	Ly6g	1A8	BD	1:200
BUV661	CD8	53-6.7	BD	1:200
BUV805	CD62L	MEL14	BD	1:200
BV480	CXCR3	CXCR3-173	BD	1:200
BV510	CD11c	N418	Biolegend	1:200
BV570	TCR beta chain	H57-597	Biolegend	1:200
BV605	Ly6c	HK1.4	Biolegend	1:200
BV650	SLAM (CD150)	TC15-12F12.2	Biolegend	1:200
BV750	CD11b	M1/70	Biolegend	1:200
BV785	PD1	29F.1A12	Biolegend	1:200
eFluor 450	FOXP3	FJK-16s	ThermoFisher	1:200
Live Dead Blue	Live/Dead	–	ThermoFisher	1:2000
PE	Tbet	4B10	Biolegend	1:200
PE-Cy7	CD64	A7R34	Biolegend	1:200
PE-Dazzle 594	CX3CR1	SA011F11	BioLegend	1:200
PE-Fire810	B220	RA3-6B2	Biolegend	1:200
PerCP	CD45.2	104	Biolegend	1:200
PerCP-eFluor 710	CD185 (CXCR5)	SPRCL5	ThermoFisher	1:200
Spark Blue 550	CD3	17A2	Biolegend	1:200
Super Bright 436	CD127	A7R34	ThermoFisher	1:200

Supplemental Table 3. List of antibodies used for flow cytometry. Flow cytometry was performed on lung tissue from young and aged mice at day 0, day 3, and day 9 post-influenza infection.
