# Supplementary material for: A spatial sequencing atlas of age-induced changes in the lung during influenza infection
Source: Nat Commun. 2023 Oct 18;14:6597. doi: 10.1038/s41467-023-42021-y (PMC10584893; doi:10.1038/s41467-023-42021-y)
Supplement: Supplementary file 2 — Reporting Summary [file 41467_2023_42021_MOESM2_ESM.pdf]

nature portfolio

Corresponding author(s): Weiguo Cui

Last updated by author(s): Sep 7, 2023

# Reporting Summary

Nature Portfolio wishes to improve the reproducibility of the work that we publish. This form provides structure for consistency and transparency in reporting. For further information on Nature Portfolio policies, see our Editorial Policies and the Editorial Policy Checklist.

## Statistics

For all statistical analyses, confirm that the following items are present in the figure legend, table legend, main text, or Methods section.

| n/a | Confirmed | |
|---|---|---|
| [ ] | [x] | The exact sample size (*n*) for each experimental group/condition, given as a discrete number and unit of measurement |
| [ ] | [x] | A statement on whether measurements were taken from distinct samples or whether the same sample was measured repeatedly |
| [ ] | [x] | The statistical test(s) used AND whether they are one- or two-sided<br>*Only common tests should be described solely by name; describe more complex techniques in the Methods section.* |
| [x] | [ ] | A description of all covariates tested |
| [x] | [ ] | A description of any assumptions or corrections, such as tests of normality and adjustment for multiple comparisons |
| [ ] | [x] | A full description of the statistical parameters including central tendency (e.g. means) or other basic estimates (e.g. regression coefficient) AND variation (e.g. standard deviation) or associated estimates of uncertainty (e.g. confidence intervals) |
| [ ] | [x] | For null hypothesis testing, the test statistic (e.g. *F*, *t*, *r*) with confidence intervals, effect sizes, degrees of freedom and *P* value noted<br>*Give P values as exact values whenever suitable.* |
| [x] | [ ] | For Bayesian analysis, information on the choice of priors and Markov chain Monte Carlo settings |
| [x] | [ ] | For hierarchical and complex designs, identification of the appropriate level for tests and full reporting of outcomes |
| [x] | [ ] | Estimates of effect sizes (e.g. Cohen's *d*, Pearson's *r*), indicating how they were calculated |

*Our web collection on statistics for biologists contains articles on many of the points above.*

## Software and code

Policy information about availability of computer code

| | |
|---|---|
| Data collection | Software was not used for data collection. |
| Data analysis | scRNA-seq - Data were demultiplexed and aligned to the mm10 2020-A reference transcriptome (10x Genomics) using Cell Ranger (v6.0, 10x Genomics). Analysis was performed in R using Seurat (v4.0) 120, with tidyverse (v1.3.1) used for data organization 121. Quality control was performed to exclude low quality cell, doublets, and dead cells; cells with more than 1000 unique genes (day 0 samples), more than 3500 unique genes (day 3 and day 9 samples), less than 200 unique genes (all samples), or greater than 10% mitochondrial genes were excluded from the final analysis. Day 3 and day 9 post-infection samples collected by our lab were then integrated with publicly available day 0 data 29 using Seurat integration pipeline. Gene set scoring was performed using Seurat’s AddModuleScore function with default settings for both scRNA-seq and spatial transcriptomics data. Receptor-ligand analysis was performed using CellChat (v1.1.3) 37 with default settings.<br><br>Bulk RNA-seq - Demultiplexing was performed using bcl2fastq (v2.20, Illumina). Alignment was performed using Salmon (v1.4.0) 122 and the GRCm39 reference genome (Ensembl) with default settings. Downstream analysis was performed in R using DESeq2 (v1.28.1) 123.<br><br>Spatial sequencing - Demultiplexing and alignment was performed with Space Ranger (v2.1, 10x Genomics) and the mm10 2020-A reference transcriptome (10x Genomics). Analysis was mainly performed in R using Seurat (v4.0). Visium datasets were integrated using the SCTransform pipeline, and PCA and UMAP were performed using the top 30 principal components. SPOTlight 33 was used to demultiplex Visium data with our integrated scRNA-seq data used as a reference. |

For manuscripts utilizing custom algorithms or software that are central to the research but not yet described in published literature, software must be made available to editors and reviewers. We strongly encourage code deposition in a community repository (e.g. GitHub). See the Nature Portfolio guidelines for submitting code & software for further information.

# Data

Policy information about availability of data

All manuscripts must include a data availability statement. This statement should provide the following information, where applicable:

- Accession codes, unique identifiers, or web links for publicly available datasets
- A description of any restrictions on data availability
- For clinical datasets or third party data, please ensure that the statement adheres to our policy

Single-cell RNA sequencing, spatial sequencing, and bulk RNA sequencing data from this paper are available in the GEO database with accession numbers GSE202325 [https://www.ncbi.nlm.nih.gov/geo/query/acc.cgi?acc=GSE202325], GSE202322 [https://www.ncbi.nlm.nih.gov/geo/query/acc.cgi?acc=GSE202322], and GSE202324 [https://www.ncbi.nlm.nih.gov/geo/query/acc.cgi?acc=GSE202324], respectively. The flow cytometry data generated in this study are provided in the Source Data file.

# Research involving human participants, their data, or biological material

Policy information about studies with human participants or human data. See also policy information about sex, gender (identity/presentation), and sexual orientation and race, ethnicity and racism.

| | |
|---|---|
| Reporting on sex and gender | Not applicable |
| Reporting on race, ethnicity, or other socially relevant groupings | Not applicable |
| Population characteristics | Not applicable |
| Recruitment | Not applicable |
| Ethics oversight | Not applicable |

Note that full information on the approval of the study protocol must also be provided in the manuscript.

# Field-specific reporting

Please select the one below that is the best fit for your research. If you are not sure, read the appropriate sections before making your selection.

☒ Life sciences ☐ Behavioural & social sciences ☐ Ecological, evolutionary & environmental sciences

For a reference copy of the document with all sections, see nature.com/documents/nr-reporting-summary-flat.pdf

# Life sciences study design

All studies must disclose on these points even when the disclosure is negative.

| | |
|---|---|
| Sample size | For flow cytometry experiments, a sample size analysis was performed assuming alpha of 0.05, beta of 0.2, and mean values of 20% with a standard deviation of 5%. This resulted in a sample size of 4 mice per group, which is the minimum in our figures. Most of our flow cytometry experiments did, in fact, have similar values to the assumptions made for our sample size calculations (see Figure 4F as an example), so we believe our sample sizes were appropriate.<br><br>Single-cell RNA sequencing and bulk RNA sequencing n numbers were chosen to comply with de facto standards in the field, where 2-3 replicates is considered sufficient. Spatial transcriptomics were done as single experiments without duplication due to the cost associated with reagents. |
| Data exclusions | No data were excluded |
| Replication | * All attempts at replicating flow cytometry experiments were successful. In general, our lab performs flow cytometry experiments at least 3-4 times with sufficient biological replicates per experiment based on prior power analyses. |
| Randomization | N/A. Young and aged mice were all infected with the same dose of influenza virus. |
| Blinding | Blinding was not relevant as all mice were infected with the same dose of virus. |

# Reporting for specific materials, systems and methods

We require information from authors about some types of materials, experimental systems and methods used in many studies. Here, indicate whether each material, system or method listed is relevant to your study. If you are not sure if a list item applies to your research, read the appropriate section before selecting a response.

## Materials & experimental systems

| n/a | Involved in the study |
|---|---|
| | x Antibodies |
| x | Eukaryotic cell lines |
| x | Palaeontology and archaeology |
| | x Animals and other organisms |
| x | Clinical data |
| x | Dual use research of concern |
| x | Plants |

## Methods

| n/a | Involved in the study |
|---|---|
| x | ChIP-seq |
| | x Flow cytometry |
| x | MRI-based neuroimaging |

# Antibodies

Antibodies used

Color Marker Clone Manufacturer Lot# Catalog# Dilution
AF594 CD4 GK1.5 Biolegend B359565 100446 1:200
AF647 TCR gamma delta GL3 Biolegend B326782 118134 1:200
APC H2Db PA tetramer NIH 58673 58673 1:100
APC-Cy7 SiglecF E50-2440 BD 2080885 565527 1:200
BUV496 CD44 IM7 BD 2049753 741057 1:200
BUV615 Ly6g 1A8 BD 2109327 751263 1:200
BUV661 CD8 53-6.7 BD 2049746 750023 1:200
BUV805 CD62L MEL14 BD 2049748 741924 1:200
BV480 CXCR3 CXCR3-173 BD 2049749 746651 1:200
BV510 CD11c N418 Biolegend B338021 117337 1:200
BV570 TCR beta chain H57-597 Biolegend B348010 109231 1:200
BV605 Ly6c HK1.4 Biolegend B335982 128035 1:200
BV650 SLAM (CD150) TC15-12F12.2 Biolegend B326904 115931 1:200
BV750 CD11b M1/70 Biolegend B348164 101267 1:200
BV785 PD1 29F.1A12 Biolegend B337077 135225 1:200
eFluor 450 FOXP3 FJK-16s ThermoFisher 2295642 2295642 1:200
Live Dead Blue Live/Dead ThermoFisher 2356793 L34961 1:2000
PE Tbet 4B10 Biolegend B347807 644810 1:200
PE-Cy7 CD64 A7R34 Biolegend B348487 139313 1:200
PE-Dazzle 594 CX3CR1 SA011F11 BioLegend B317312 149013 1:200
PE-Fire810 B220 RA3-6B2 Biolegend B358842 103287 1:200
PerCP CD45.2 104 Biolegend B283428 109826 1:200
PerCP-eFluor 710 CD185 (CXCR5) SPRCL5 ThermoFisher 2403331 46-7185-80 1:200
Spark Blue 550 CD3 17A2 Biolegend B315668 100259 1:200
Super Bright 436 CD127 A7R34 ThermoFisher 2308318 62-1271-82 1:200
Biotin CD3e 145-2C11 Biolegend B278768 133307 1:300
Biotin CD8a 53-6.7 Biolegend B269028 100704 1:300
Biotin TCRb H57-597 Biolegend B269722 109204 1:300
Biotin B220 RA3-6B2 Biolegend B278770 133307 1:300
Biotin NK1.1 PK136 Biolegend B281631 108704 1:300
Biotin Ly6c/g RB6-8C5 Biolegend B278769 133307 1:300
Biotin CD11b M1/70 Biolegend B278766 133307 1:300
Biotin CD11c N418 Biolegend B270359 117304 1:300
Biotin Ter119 TER-119 Biolegend B278765 133307 1:300
Fixable Violet Live/Dead N/A ThermoFisher N/A L34963 1:1000
PerCP-Cy5.5 CD90 OX-7 Biolegend B256437 202516 1:200

Validation

* All antibodies used in this manuscript are commonly used and were validated commercially by Biolegend, BD, and ThermoFisher. Antibody clone numbers, dilutions, and manufacturer names are provided in a table in the Methods section of the manuscript. No newly developed or unvalidated antibodies were used in this manuscript.

# Animals and other research organisms

Policy information about studies involving animals; ARRIVE guidelines recommended for reporting animal research, and Sex and Gender in Research

Laboratory animals

Young (16- to 18-week-old) and aged (80- to 82-week-old) female C57Bl/6J mice were purchased from Jackson. Mice were housed in a room set to a 14/10 light/dark cycle. The average temperature was 69 degrees Fahrenheit with an average humidity of 46% in the cooler months and 70% in the summer.

Wild animals

No wild animals were used in this study.

| | |
|---|---|
| Reporting on sex | Only female mice were used. |
| Field-collected samples | No field-collected samples were used in this study. |
| Ethics oversight | All mice were maintained under the guidelines of the Institutional Animal Care and Use Committees (IACUC) of the Medical College of Wisconsin (MCW). |

Note that full information on the approval of the study protocol must also be provided in the manuscript.

# Flow Cytometry

## Plots

Confirm that:

- [x] The axis labels state the marker and fluorochrome used (e.g. CD4-FITC).
- [x] The axis scales are clearly visible. Include numbers along axes only for bottom left plot of group (a 'group' is an analysis of identical markers).
- [x] All plots are contour plots with outliers or pseudocolor plots.
- [x] A numerical value for number of cells or percentage (with statistics) is provided.

## Methodology

| | |
|---|---|
| Sample preparation | For flow cytometry, mice were euthanized and the right ventricle was perfused with 10 mL cold DPBS (Corning). Lungs were minced into fine pieces and placed into a 50 mL tube containing 20 mL of 1.3 mM EDTA in Hank's Balanced Salt Solution, which was supplemented with 12 mg/mL HEPES sodium salt, 2.9 mg/mL L-glutamine powder, 0.2x penicillin-streptomycin solution, and 0.05 mg/mL gentamycin sulfate (1x HGPG). After shaking for 30 minutes at 37 °C, the 20 mL supernatant was carefully removed from the minced lung tissue and 25 mL of 1% FBS in cRPMI supplemented with 1 mM CaCl2, 1 mM MgCl2, 2500 units of collagenase I, and 1x HGPG was added to lung tissue. This was placed on a shaker for 60 minutes at 37 °C. The residual tissue was homogenized in a 70 µm cell strainer. The suspension was centrifuged for 10 minutes at 1500 rpm. Supernatant was removed and the cell pellet was suspended in 4 mL of 44% Percoll (in 1% cRPMI), and transferred to a 14 mL round bottom tube. Then, 2.5 mL of 67% Percoll (in PBS) was carefully underlaid, and this was centrifuged for 30 minutes at 1800 rpm at room temperature with no braking. Cells were pipetted out and red blood cell lysis was then performed. Live/Dead cell staining was performed using Fixable Live Dead Blue (ThermoFisher) in PBS at 4 °C for 30 minutes in the dark. Cells were washed twice and surface panel was applied to cells, incubated at room temperature for 60 minutes in the dark. Cells were fixed using 1x TrueNuclear Fix (Biolegend), and intracellular transcription factor staining was performed using TrueNuclear Perm Buffer (Biolegend) at room temperature for 45 minutes in the dark. Cells were resuspended in FACS buffer prior to analysis by flow cytometry.<br><br>For scRNA-seq, mice were euthanized and the right ventricle was perfused with 10 mL cold DPBS (Corning). The lung lobes were cut into fine pieces and digested at 37 °C with collagenase IV (1 mg/mL; Worthington) for 40 minutes. The tissue pieces were further ground and homogenized in a 70 µm cell strainer. After spinning down, red blood cells were removed by addition of ACK lysis buffer (Lonza) for 10 minutes on ice. Cells were kept in 10% RPMI (Lonza) at all times. Only day 9 post-infection samples were sorted for scRNA-seq to obtain a roughly equal proportion of immune and non-immune cells for analysis, although all samples were stained to examine the relative proportions of immune and non-immune cells. Single-cell suspensions were incubated with biotin-conjugated antibodies targeting the following Lineage markers for 30 minutes at 4 °C in 10% RPMI: CD3e, CD8a, TCRb, B220, NK1.1, Ly6C/G (anti-GR1), CD11b, CD11c, Ter119 (Biolegend). Samples were then washed twice in 10% RPMI and stained with Live/Dead Fixable Violet (ThermoFisher), streptavidin, and anti-CD90 for 30 minutes at 4 °C in 10% RPMI. Samples were then run on an Aria IIIu cell sorter (BD Biosciences). |
| Instrument | For flow cytometry, single color compensations and cellular flow cytometry were acquired and unmixed on a 5 laser spectral flow cytometer (Cytek Aurora).<br><br>For FACS sorting, samples were run on an Aria IIIu cell sorter (BD Biosciences). |
| Software | For flow cytometry, data were analyzed using Cytobank Premium (Beckman Coulter).<br><br>For FACS sorting, data were analyzed live on the instrument using FACS Diva. |
| Cell population abundance | Post-sort purity was determined by rerunning sorted samples. |
| Gating strategy | Flow cytometry: Debris gated out, live cells gated on. Then gated on TCRb/CD3e. T cells gated as double positive, then divided by CD4 and CD8. B cells gated as double negative, then as B220+. Neutrophils gated as double negative, then CD11b+ Ly6G+. Positive gates were set at approximately 10^4.<br><br>FACS sorting: Debris gated out using FSC/SSC. Live cells gate on. Sorting was then done on Lineage+ and Lineage– cells. CD90 was included for visualization but not used for sorting. |

- [x] Tick this box to confirm that a figure exemplifying the gating strategy is provided in the Supplementary Information.
